# The First AAU International Conference on Pharmacy and Biomedical Sciences

**DOI:** 10.1186/s12919-023-00276-9

**Published:** 2023-10-05

## Oral Presentations

### O1 A stable conformer may lead to the chirality without an apparent chiral center; a model through a bis-bromoindole derivative as candidate drug against some serotonin receptors

#### Kamal Sweidan^1^, Muhammed Alzweiri^2^, Firas Awwadi^1^

##### ^1^Department of Chemistry, School of Science, The University of Jordan, Amman, Jordan; ^2^Faculty of Pharmacy, University of Jordan, Amman, Jordan

###### **Correspondence:** Muhammed Alzweiri (m.alzweiri@ju.edu.johe)

*BMC Proceedings 2023*, **17(16):**O1

Background

Biscompounds may give better chance of binding with receptors due to increasing the probability of attachment from two sides of the structure with the receptor. On the other hand, the chirality introduces a chance for further adjustment of binding.

Materials and methods

3,3'-(2-nitropropane-1,3-diyl)bis(4-bromo-1H-indole) has been synthesized as a model compound with intentional modifications via bromine atoms in order to give rise a possibility of variation in dipole moment around the stable conformer of the structure. Consequently, this may induce the chirality in the compound even without an apparent chiral center. NMR and single X-ray crystallography were used to study the chirality of the compound. Subsequently, the generated isomers were exposed for docking studies against a group of common serotonin receptors.

Results and conclusions

The chirality of the biscompound was confirmed by the analytical tools. Interestingly, its optical isomerism was discriminated by some of the receptors including 5HT3A, 5HT2B-bril, 5HT1F and 5HT1A. The docking scores of the biscompound isomers are not very inferior from those of the positive control; serotonin. Interestingly, some fine modifications such as the reduction of the nitro group in the biscompound generates a candidate compound has even more affinity toward the tested receptors than the positive control, except against 5HT1F.

### O2 Teratogenic potential of Solenstemma argel extract in Wistar albino rats

#### Nazik M.E Muatafa^1^, Shahenaz Satti^2^, Nafisa Osman^3^, Ahmed A Gameel^4^, Tarig M El-Hadiyah^5^

##### ^1^Department of Pharmacology, Faculty of Pharmacy, Al Neelain University, Khartoum, Sudan; ^2^Department of Physiology, Faculty of Medicine, Al Neelain University, Khartoum, Sudan; ^3^Department of Pharmaceutical Chemistry, Faculty of Pharmacy, Al Neelain University, Khartoum, Sudan ^4^Department of Pathology, Faculty of Veterinary Medicine, University of Khartoum, Khartoum, Sudan; ^5^Department of Pharmacology, Faculty of Pharmacy, International University of Africa, Khartoum, Sudan

###### **Correspondence:** Nazik M.E Muatafa (nazik.mohamed@neelain.edu.sd)

*BMC Proceedings 2023*, **17(16):**O2

Most people in Africa use herbal remedies for their primary healthcare needs. Herbal medicine may permanently harm a fetus' development.

The teratogenic potential of Solenstemma argel extract was investigated in this study in pregnant Wister albino rats. A dose of 250 mg/kg Solenstemma argel extract was administered to pregnant rats intraperitoneal from the seventh to the sixteenth day of gestation. The group receiving Solenstemma argel extract displayed 25% of the fetuses abnormal. Fetal abnormalities including body bleeding, limb deformities, and fetus resorption. That was significantly different from the control group (P-value = 0.01). Furthermore, histopathological findings of liver sections from fetuses of Solenstemma argel - treated mothers showed loose liver texture and hepatocytes hemorrhage.

Based on the findings of this study, we conclude that administration of Solenstemma argel extract to pregnant rats during the organogenesis phase have teratogenic effects and alter liver histology of fetuses.

### O3 Lipid nanoparticles formulations: from bench scale to industrial scale

#### Mohammad A Obeid

##### Department of Pharmaceutics and Pharmaceutical Technology, Faculty of Pharmacy, Yarmouk University, Irbid, Jordan

###### **Correspondence:** Mohammad A Obeid (m.obeid@yu.edu.jo)

*BMC Proceedings 2023*, **17(16):**O3

Lipid nanoparticles are self-assembling vesicles obtained by hydrating a mixture of non-lipids and cholesterol and are suitable as carriers of drugs and biopharmaceuticals. It is desirable to be able to accurately control size and polydispersity of the vesicles as this can impact on biological outcomes. Moreover, its crucial to formulate these nanoparticles in a scalable method that can be used in industrial settings. One approach that has been successful for lipid-based systems is the use of microfluidic mixing which allows for the precise control of the generated nanoparticles. Microfluidic mixing has been compared with a traditional method such as thin film hydration method and heating method using niosomes as a model nanoparticle.

Niosomes are lipid bilayer vesicles that are composed of non-ionic surfactant. These nanoparticles were successfully prepared by microfluidic mixing which is a recently developed method used to prepare lipid-based nanoparticles and results in the production of small vesicles with efficient encapsulation of a therapeutic agent. To prepare niosomes using microfluidic mixing, specific volumes from each stock solution of the lipids components will be mixed together to prepare the lipid phase. The lipid phase will then be injected into the first inlet and the aqueous phase into the second inlet of the microfluidic microchannel, with the mixing temperature set above the phase transition of the lipids. The flow rate ratios (FRR) between the aqueous and organic phase and the total flow rates (TFR) of both phases are among the factors that control the particles production using this method.

The generated niosomes were compared with the niosomes that have the same components but prepared with different methods such as the thin film hydration method which usually will be followed by extrusion step for size reduction.

The size of niosomes produced by microfluidic mixing was controlled by altering the FRR and TFR in both the lipid and aqueous phases. In contrast, niosomes prepared by the TFH method and heating method were large, polydisperse, and required a post-manufacturing extrusion size reduction step (around 4μm ± 0.2 before extrusion). A stability study for the prepared niosomes at four temperatures (4, 25, 37 and 50°C) for 4 weeks indicates that the vesicles were shown to be stable in terms of size and polydispersity index (PDI).

The prepared particles were investigated for their ability to encapsulate and deliver several therapeutic agents such as doxorubicin, paclitaxel, cisplatin, nucleic acids, and many other hydrophilic and hydrophobic molecules.

### O4 Antioxidant and α-amylase inhibitory activities of different extracts of olive leaves from Jordan

#### Maher M Al-Dabbas

##### College of Pharmacy, Department of Nutrition and Dietetics, Al Ain University, Abu Dhabi, UAE

###### **Correspondence:** Maher M Al-Dabbas (maher.dabbas@aau.ac.ae)

*BMC Proceedings 2023*, **17(16):**O4

The present study was designed to evaluate the antioxidant and α-amylase inhibitory activities of aqueous, ethanol and ethyl acetate extracts from Nabali and wild olive leaves grown in Jordan. The extracts were procured through ultrasonic-assisted extraction. Three experimental models were employed for the antioxidant activity evaluation of each extract (DPPH radical scavenging activity, chelating power and reducing power activities). The enzymatic inhibitory activity of α-amylase was evaluated by CNP-G3 assay. Moreover, total phenolics, flavonoids and flavonols contents of the olive leaves extracts were quantified. The ethanolic wild leaves extract showed the highest total phenolics content (113.97 mg GAE/g), followed by the ethyl acetate extract of Nabali leaves (102.2 mg GAE/g). Flavonoid and flavonol contents were significantly (P ≤ 0.05) the highest in ethyl acetate extract of wild leaves (123.07 mg RE/g and 91.3 mg RE/g, respectively). The ethanolic wild leaves extract and ethyl acetate extract of Nabali leaves showed the highest DPPH scavenging activity with IC50 value of 192.1 μg/ml. The total antioxidant activity was found to be the highest in ethanolic wild leaves and ethyl acetate of Nabali leaves extracts (202.1 and 202.3 μg ascorbic acid equivalent for 1mg extract, respectively). The ethanolic, ethyl acetate wild leaves extracts and ethyl acetate of Nabali extract at concentration of 100 μg/ml showed the highest chelating activity of ferrous ions (52.4, 50.5 and 47.2 %, respectively). The ethanolic extracts of wild and Nabali leaves showed the highest inhibitory activity against α-amylase from the porcine pancreas by 65.1% and 62.3%, respectively at concentrations of 10 mg/ml. All extracts showed remarkable antioxidant activities determined with different methods in a dose dependent manner and the effects depend strongly on the solvent used for the extraction.

### O5 A Promising avenue for Raloxifene as an anticancer agent: comparative nanovesicles formulation, characterization, and in vitro cytotoxicity study

#### Jana K Al Wattar, Mohammad Assi, Mohammad Rahal

##### Department of Pharmaceutical Sciences, Faculty of Pharmacy, Lebanese International University, Beirut, Lebanon

###### **Correspondence:** Jana K Al Wattar (Jana.wattar@liu.edu.lb)

*BMC Proceedings 2023*, **17(16):**O5

Background

Triple negative breast cancer is an aggressive disease, which accounts for high percentage of breast cancer morbidity, hence rigorous efforts are focused on the development of effective therapies to overcome the disorder. Raloxifene hydrochloride (RLX) is an FDA approved, second generation, selective estrogen receptor modulator, which acts as an antiresorptive agent in bones which increases bone mineral density thus it is used in the treatment of osteoporosis. In addition, the antiandrogenic and antiestrogen activities of RLX allow its application for long term female hormonal replacement therapy, fibrocystic disease and benign prostate hypertrophy. Furthermore, RLX can be used in postmenopausal women for treatment of breast cancer. Though, this drug possesses the desirable therapeutic activity and minimized risk aptitude, yet its pharmacokinetic characteristics represent a drawback for its use. To overcome these limitations, a series of nanovesicles as a potential anticancer targeting system were investigated.

Materials and methods

Raloxifene loaded nanocarriers including hexosomes, nanoliposomes, nanoniosomes and nanomicelles were formulated and characterized including particle size, zeta potential, morphology, entrapment efficiency, structural elucidation and cytotoxicity.

Results

Results revealed that each nanocarrier presented a nanometric size with reasonable encapsulation efficiency yet the RLX loaded hexosomal formulation exhibited the lowest mean particle size of 96.0 ± 3.1 nm with narrow distribution, highest entrapment efficiency (92.6±5.3%.) and lowest zeta potential (33.6±2.2 mV) confirming the effectiveness of this lipidic nanosystem for RLX encapsulation. Cytotoxicity of the different Raloxifene loaded nanovesicles on MCF-7 breast cancer cell lines and MCF10 non tumorigenic cell revealed the substantial cytotoxic activity of the hexosomal nanocarrier compared to the other nanovesicles exhibiting the lowest IC50 = 57.5 μm.

Conclusion

Cell viability in both breast cancer cell lines confirmed the effective cytotoxicity of loaded hexosomal nanocarrier with a dose-dependent decrease. Thus future subcellular and molecular studies of this effective nanocarrier system must be performed.

### O6 Fortified blended foods from extruded sorghum cowpea and sorghum soybean blends

#### Sajid Alavi^1^, Nicole Delimont^1^, Sirichat Chanadang^2^, Michael Joseph^3^, Brian Lindshield^1^, Edgar Chambers^1^, Mahmoud Abu-Ghoush^4^

##### ^1^Kansas State University, Manhattan, Kansas, USA; ^2^Srinakharinwirot University, Bangkok, Thailand; ^3^North Carolina State University, Raleigh, North Carolina, USA; ^4^Al Ain University, Al Ain, United Arab Emirates

###### **Correspondence:** Sajid Alavi (salavi@ksu.edu)

*BMC Proceedings 2023*, **17(16):**O6

Background

Fortified blended foods (FBFs) are the primary products used by food aid agencies in alleviating undernutrition around the world [1].

Materials and Methods

New FBFs based on grain sorghum were developed using extrusion. Binary mixtures of cowpea flour or soybean flour and sorghum flour were extruded to produce sorghum cowpea and sorghum soy composites. Extrudates were ground using a hammer mill and fortified with nutrients to obtain sorghum cowpea and sorghum soy blends. A 20-week long clinical nutrition trial involving 2,000 children of age 6-60 months was conducted in Tanzania to study the efficacy of the FBFs.

Results

The products met the new international nutritional guidelines for FBFs including energy content of 400 kcal/100g, protein content of 18% and an adequate complement of micronutrients such as iron and vitamin A. Physicochemical properties of the extruded and ground composites were related to their final quality including flowability and ease of consumption. Odds for anemia occurrence decreased faster with all tested FBFs as compared to the control group, with greatest impact observed for sorghum cowpea blend. After 20 weeks, extruded sorghum based fortified blend had the highest odds against vitamin A deficiency and this was the only cluster showing a statistically significant (p<0.05) improvement in comparison to the control cluster. Consumer testing results indicated that all new FBFs were more preferred by Tanzanian children as compared to traditional non-extruded FBF (corn soy blend plus).

Conclusions

The newly developed extruded sorghum-cowpea and sorghum-soy FBFs were found to be suitable alternatives to the traditional corn-soy blend product used in food aid [2]. These products can broaden the basket of commodities in nutritional intervention programs, have the potential to be GMO-free, can address price volatility and allow easier local and regional procurement as crops such as sorghum and cowpea are grown indigenously in Africa.

Trial registration

ClincialTrials.gov Identifier NCT02847962

Acknowledgements

McGovern-Dole International Food for Education and Child Nutrition Program, Micronutrient Fortified Food Aid Products Pilot (MFFAPP) United States Department of Agriculture (USDA) Foreign Agriculture Service (FAS).

References

1. Joseph. Extrusion, Physico-chemical characterization and nutritional evaluation of sorghum-based high protein, micronutrient fortified blended foods. M.V. 2016; Ph.D. thesis, Kansas State University.
2. Delimont, N.M., Vahl, C.I., Kayanda, R., Msuya, W., Mulford, M., Alberghine, P., Praygod, G., Mngara, J., Alavi, S., and Lindshield,. Complementary feeding of sorghum-based and corn-based fortified blended foods results in similar iron, vitamin A and anthropometric outcomes in the MFFAPP Tanzania efficacy study. B.L. 2019; Current Developments in Nutrition, nzz027.

### O7 Effect of magnesium supplementation on diabetic peripheral neuropathic pain: a randomized, placebo-controlled study of diabetic patients with hypomagnesemia

#### Nadia Hussain^1,2^, Amal Hussain Ibrahim AlHaddad^3^, Semira Beshir^4^, Zainab Khan^5^, Amira S.A Said^6,7,^

##### ^1^Department of Pharmaceutical Sciences, College of Pharmacy, Al Ain University, UAE; ^2^AAU Health and Biomedical Research Center, Al Ain University, Abu Dhabi, UAE; ^3^Chief Operations Office, Sheikh Shakhbout Medical City (SSMC) in partnership with Mayo Clinic, Abu Dhabi, UAE; ^4^Clinical Pharmacy & Pharmacotherapeutics Department, Dubai Pharmacy College, Dubai, UAE; ^5^Department of Internal Medicine, Punjab Care hospital, Lahore, Punjab, Pakistan; ^6^Department of Clinical Pharmacy, College of Pharmacy, Al Ain University, Al Ain, UAE; ^7^Clinical Pharmacy Department, Faculty of Pharmacy, Beni Suef University, Beni Suef, Egypt

###### **Correspondence:** Nadia Hussain (nadia.hussain@aau.ac.ae)

*BMC Proceedings 2023*, **17(16):**O7

Diabetic peripheral neuropathy that affects approximately 60% of individuals with diabetes worldwide with significant impact of patient’s quality of life. Hypomagnesemia is associated with the development of neuropathy. In the present study we investigated the effect of magnesium supplementation on diabetic neuropathic pain and peripheral nerve function over 24 weeks in South Asian male diabetic patients with established peripheral diabetic neuropathy and hypomagnesemia. Analgesic effectiveness was assessed by observing any change in the Numeric Pain Rating Scale (NPRS) score, Brief Pain Inventory (BPI) for painful diabetic peripheral neuropathy (BPI-DPN question 4) and Patient Global Impression of Change (PGIC). Demographic, medical and laboratory data including serum magnesium and HbA1c were collected. Our study screened 212 male patients with T2DM and 63 had hypomagnesemia. These individuals were then divided into two groups; those who received magnesium supplementation (Group M) and those who received placebo (Group PM). Group M experienced a more significant reduction in the average pain intensity (p < 0.05) during the last 24 hours. Group M showed more significant reduction of pain compared to the control group (p <0.01), a baseline score of 5.1 ± 1.2 dropped to 3.1 ± 1.5 by week 24 of treatment, in comparison to control group. Change in mean daily pain intensity was – 2.3 ± 1.5 [95% CI: -2.45, -1.5]. Group M experienced a significant overall improvement in the health status during the study. After week 2 of treatment, patient satisfaction scores were 2.8 ± 1.1 in Group M which increased to 3.9 ± 1.2 by week 24 of treatment. Our study showed a sustained response to magnesium supplementation as evidenced by the maintenance of treatment response over 24 weeks. Magnesium supplementation was generally well tolerated and an effective long-term treatment option that may additionally benefit those patients requiring pain relief from systemic therapies.

### O8 Innovative strategy to reveal the unique chemistry of the human microbiome

#### Walaa Mousa^1,2^

##### ^1^College of Pharmacy, Al Ain University, Al Ain, UAE; ^2^College of Pharmacy, Mansoura University, Mansoura, Egypt

###### **Correspondence:** Walaa Mousa (walaa.mousa@aau.ac.ae)

*BMC Proceedings 2023*, **17(16):**O8

Background

The human body hosts trillions of diverse microbes, collectively known as the microbiota. These microbes secrete molecules with unique chemistry thought to be evolved with us to specifically recognise human cellular receptors resulting in health or disease. Discovery of these unique chemistry will revolutionize our understanding of the microbiome-host interaction and represent an opportunity for translational research and commercialization. Current discovery approach relies mostly on machine learning tools that predict the gene cluster encoding specific classes of secondary metabolites. These genome-centered tools predict molecules based on similarity to known compounds and genes meaning they can only predict known classes of natural products and fail to reveal the entire landscape of microbiome-secreted chemistry.

Methods

In this research, we use a mass spectrometry-based protocol to identify novel ions based on molecular networking strategy.

Results

Using this analysis we report the discovery of two unique microbiome-molecules with immunomodulatory activity.

Conclusion

This research leverages our understanding of microbiome-chemistry and its impact on developing microbiome-based therapeutics.

### O9 Development and validation of green ATR-FTIR spectroscopic method for quantitative analysis of ibuprofen tablets

#### Khairi M. S Fahelelbom, Abdullah Salah, Ramez Mansour, Rami Abujarad

##### Department of Pharmaceutical Sciences, College of Pharmacy, Al Ain University, P.O. Box 64141, Al Ain, United Arab Emirates

###### **Correspondence:** Khairi M. S Fahelelbom (Khairi.mustafa@aau.ac.ae)

*BMC Proceedings 2023*, **17(16):**O9

Background

Attenuated total reflection- Fourier transform infrared (ATR-FTIR) spectroscopy has been successfully employed as a quantitative approach for the determination of Ibuprofen in some of its commercially available dosage forms. Herein, the technique has been validated as an alternative green tool that evades necessary sample preparation procedures required in traditional quantitative methods

Method

The CO of the Ibuprofen stretching band in the range 1620-1750 cm-1 has been selected for the quantitative determination of Ibuprofen in original samples. The first derivative measurements have determined the packing area (AUC) from ATR-FTIR spectral scanning of samples. Spectral data analysis and method validation including linearity, specificity, the limit of detection, accuracy, and precision were determined. The technique has been validated through the comparison of statistical results according to the standard procedures.

Objective

Green and nondestructive method using the ATR-FTIR method was proposed and validated for the quantitative analysis of Ibuprofen tablet dosage forms

Results

Assay tests have indicated that there are no excipients or additives of the commercial tablets interferences. The linearity is excellent within the concentration range of 0.2 to 1.5 w/w % (r = 0.9994). Repeated analysis results are associated with comparable standard and relative standard deviations. Hence, it is concluded that the precision of the technique is acceptable. A percentage of recoveries of 99.81, 101.54, and 99.41 respectively are in good agreement with pharmacopeia percent recovery standards. The high degree of sensitivity of the technique was demonstrated by obtaining a 0.028 w/w % detection limit and a 0.1599 w/w % limit of quantification values.

Conclusions

The first derivative ATR-FTIR spectroscopy has been successfully demonstrated as a greener alternative method in pharmaceutical analysis. The high accuracy and precision degrees obtained in this method for a number of commercial Ibuprofen tablets can be generalized for many similar applications.

### O10 Urinary tract infection case study

#### Adel S Sadeq

##### College of Pharmacy, Al Ain university, Al Ain Campus, Al Ain, Abu Dhabi, UAE

###### **Correspondence:** Adel S Sadeq (adel.sadeq@aau.ac.ae)

*BMC Proceedings 2023*, **17(16):**O10

Urinary tract infection (UTI) is the most common bacterial infection found in both males and females after respiratory and gastro-intestinal infections.

Despite the fact, that both the genders are susceptible to the infection, women are mostly vulnerable due to their anatomy and reproductive physiology.

The infection is usually caused as a consequence of bacterial invasion of the different parts of the urinary tract including the lower and the upper urinary tract. UTIs is the most common cause of both community-acquired and nosocomial infections for patients admitted to hospitals.

The repeated infections by an organism from the same species that caused previous infections is typically responsible for recurrences. For better management and prognosis, it is mandatory to know the possible site of infection, whether the infection is uncomplicated or complicated, re-infection or relapse, or treatment failure and its pathogenesis and risk factors.

Symptomatic urinary tract infections occur most commonly in women of child-bearing age. There was an association between ESBL causing UTIs and the BMI, recent hospitalization, and recent antibiotics usage and diabetes mellitus.

Identifying the risk factors of UTIs caused by ESBL bacteria helps to determine the high-risk patients and enables the most appropriate antimicrobial treatment. Cystitis predominates but needs to be distinguished from acute urethral syndrome that affects both sexes and has a different management plan than UTIs. Complicated urinary tract infection case study is presented as one example.

### O11 A Bibliometric study: Pharmacy practice research in the UAE

#### Daneh Obaid^1^, Faris El-Dahiyat^1^, Zaheer-Ud-Din Babar^2^

##### ^1^Clinical Pharmacy Program, College of Pharmacy, Al Ain University, P.O Box 64141, Al Ain, United Arab Emirates; ^2^Department of Pharmacy, School of Applied Sciences, University of Huddersfield, Huddersfield HD1 3DH, West Yorkshire, UK

###### **Correspondence:** Daneh Obaid (dana.obaid@aau.ac.ae)

*BMC Proceedings 2023*, **17(16):**O11

Introduction

The importance of developing research activity in the pharmacy practice domain was emphasized by the proactive changes that occur in this field.[1] Evidence from pharmacy practice research validates the necessity and effectiveness of current and potential pharmacy services.[2] The UAE is from the high-income countries in the middle east that provide a pronounced intention for scientific research.[3] This review is designed to identify the pharmacy practice literature published in the UAE.

Method

A bibliometric analysis was conducted based on previously collected data about Pharmacy practice and clinical pharmacy research in the Middle East.[4] A thematic synthesis was used to visualize the themes covered in the UAE publications.

Results

82 publications were identified in the field of pharmacy practice in the UAE during the period between 2009-2019. Around 70% of these publications were on two themes; Pharmacy practice and pharmacist services with n=31 (37.8%) articles, and Medication use and pharmacogenomics with n=26 (31.7%) articles. Less intensity of studies was about Medication safety and pharmacovigilance n=7 (8.5%), and Pharmacy education and professional development n=10(12.2%). Furthermore, a lack of studies concerning; Medication information and public health promotion n=2 (2.4%), Pharmaco-economics and pharmaceutical policies n=2 (2.4%), and Clinical research n=4 (5%) was noticed.

Conclusion

The predominant theme observed in UAE for pharmacy practice research articles was Pharmacy practice and pharmacist services, however, a deficiency in pharmaco-economic and pharmaceutical policies studies was detected. Some repetitive ideas were noticed in several themes; this can be avoided by setting a research agenda.

References

1. Hasan SS, Thiruchelvam K, Kairuz T, Abbas N, Babar ZU. Pharmacy practice and its research: evolution and definitions. Encyclopedia of Pharmacy Practice and Clinical Pharmacy. Elsevier-Academic Press; 2019 Jan
2. Luetsch K, Maidment I, Twigg M, Rowett D. Realist research to inform pharmacy practice and policy. Research in social and administrative pharmacy. 2021 Dec 1;17(12):2075-81.
3. Meo SA, Usmani AM, Vohra MS, Bukhari IA. Impact of GDP, spending on R&D, number of universities and scientific journals on research publications in pharmacological sciences in Middle East. Eur Rev Med Pharmacol Sci. 2013 Oct 1;17(20):2697-705.
4. Obaid D, El-Dahiyat F, Babar ZU. Pharmacy practice and clinical pharmacy research in the Middle East: a scoping review of studies from Bahrain, Iraq, Jordan, Kuwait, Lebanon, Oman, Palestine, Qatar, Saudi Arabia, Syria, United Arab Emirates, and Yemen. Journal of Pharmaceutical Policy and Practice. 2022 Dec;15(1):1-5.

### O12 Characterization of probiotic lactic acid bacteria form honeybee for potential use in food applications

#### Mohamed G Shehata^1,2^, Nourhan M Abd El-Aziz^2^, Amira M. G. Darwish^2^, Sobhy A El-Sohaimy^2,3^, Saad H Masry^4,5^

##### ^1^Food Research Section, R&D Division, Abu Dhabi Agriculture and Food Safety Authority (ADAFSA), Abu Dhabi P.O. Box 52150, United Arab Emirates; ^2^Food Technology Department, Arid Lands Cultivation Research Institute (ALCRI), City of Scientific Research and Technology Applications (SRTACITY), New Borg El-Arab City, Alexandria, Egypt; ^3^Department of Technology and Organization of Public Catering, Institute of Sport Tourism and Services, South Ural State University, Chelyabinsk 454080, Russia; ^4^Abu Dhabi Agriculture and Food Safety Authority, Al Ain, United Arab Emirates; ^5^Department of Plant Protection and Molecular Diagnoses, Arid Lands Cultivation Research Institute, City of Scientific Research and Technological Applications, 21934 Alexandria, Egypt

###### **Correspondence:** Saad H Masry (saad.masry@ADAFSA.GOV.AE)

*BMC Proceedings 2023*, **17(16):**O12

Lactic acid bacteria (LAB) are gram-positive bacterial strain which had an important role in food applications. They produce a large number of metabolites with beneficial effects on human health as a fermentation end product. This study aim to determine the probiotic characteristics and fermentation profile of eight selected LAB isolated from honeybees’ stomach and mid-gut. Physiological properties, cell surface properties (hydrophobicity, autoaggregation, co- aggregation, adhesion to Caco-2 cell), acid and bile tolerance, exopolysaccharide (EPS) production, hemolytic and tolerance to sodium chloride, resistance toward lysozyme and heat, and fermentation profile (pH and growth) were examined. The antioxidant effects of intact cells, intracellular and cell-free supernatant (CFS) of LAB strains were assessed by several antioxidant DPPH and ABTS radical scavenging assays were also determined. The results showed that all LAB isolates showed auto-aggregation ability, good hydrophobicity ability against different organic solvents, high co-aggregation, moderate antimicrobial activity and EPS production. The survival percentages of simulated gastric and intestinal juice conditions of LAB varied greatly. Among the isolates, Pediococcus pentosaceus HBMSS2 and Lactobacillus plantarum HBMSS3 exhibited remarkable tolerance to sodium chloride and good resistance toward lysozyme and heat. Besides, they showed very promising fermentation profiles. Also, the antioxidant activity of Pediococcus pentosaceus HBMSS2 and Lactobacillus plantarum HBMSS3 bacterial lysate and CFS exhibited an excellent antioxidant capacity. The current study demonstrated the high antioxidant property of a two probiotic strains from honeybee. This may be a promising finding for future applying of these probiotics in food applications.

### O13 Detoxification role of Lactobacilli fermented Opuntia ficus indica juice on cadmium toxicity in male rats

#### Mohamed G Shehata^1,2^, Nourhan M Abd El-Aziz^1^, Ahmed Noah Badr^3^, Ebtehal A Farrage^4^, Amira M G Darwish^1,5^

##### ^1^Food Technology Department, Arid Lands Cultivation Research Institute, City of Scientific Research and Technological Applications (SRTA-City), New Borg El-Arab, Alexandria 21934, Egypt; ^2^Food Research Section, R&D Division, Abu Dhabi Agriculture and Food Safety Authority (ADAFSA), Abu Dhabi 20602, United Arab Emirates; ^3^Food Toxicology and Contaminants Department, National Research Centre, Dokki, Cairo 12622, Egypt; ^4^Pathology Department, Medical Research Institute, Alexandria University, Egypt; ^5^Food Industry Technology Program, Faculty of Industrial and Energy Technology, Borg Al Arab Technological University (BATU), Alexandria 21934, Egypt

###### **Correspondence:** Mohamed G Shehata (mohamed.shehata@adafsa.gov.ae)

*BMC Proceedings 2023*, **17(16):**O13

Cadmium is well-known toxic metal capable of having adverse effects on the liver and kidney most especially on prolonged exposure. This study seeks to produce a Lactobacilli fermented Opuntia ficus indica juice and assess its detox effect on cadmium intoxicated male rats. Adult male albino rats were exposed to cadmium for 60 days at a concentration of 10 mg/kg CdCl2. Samples of blood and tissue were assayed for cadmium adverse exposure effects. Liver and kidney homogenates were used for biochemical analysis and the estimation of the expression levels of TNF-α, P53, BCL-2 and IL-1 genes. Samples of liver and kidney were also used for histological analysis. Results show that accumulation of cadmium in the liver was 4.7 folds greater than kidney. Significant (P < 0.05) alterations in the levels of TNF-α, P53, BCL-2 and IL-1 genes were observed in treated groups compared to the control. Treatment with non-fermented (NCJ) and fermented (FCJ) cactus pear juice, succeeded to exert decrease the toxic effects to the Cd-treated rats. The FCJ expressed beneficial treatment on reversing the harmful effect of Cd exposure than NCJ in experimental rats. The present study has shown that the toxicity of Cd can be ameliorated by using of non-fermented (NCJ) and fermented (FCJ) cactus pear juice and special attention should be drawn to Lactobacilli fermented products to face the heavy metals environmental pollution.

### O14 Innovative applications of Damas (Conocarpus spp.) extracts as a safe source of phenolic compounds

#### Hanan S Afifi^1^, Mohamed G Shehata^1,3^, Mahmoud Abdul Aziz^2^, Ahmed Zaki^2^, Majduleen M A Shanik^1^

##### ^1^Food Research Section, R&D Division, Abu Dhabi Agriculture and Food Safety Authority (ADAFSA), UAE; Research Station Section, R&D Division, Abu Dhabi Agriculture and Food Safety Authority (ADAFSA), UAE; ^3^Food Technology Department, Arid Lands Cultivation Research Institute (ALCRI), City of Scientific Research and Technology Applications (SRTACITY), New Borg El-Arab City, Alexandria, Egypt

###### **Correspondence:** Hanan S Afifi (hanan.afifi@adafsa.gov.ae)

*BMC Proceedings 2023*, **17(16):**O14

This study aims to discover innovative applications employing allelopathic potential of damas (Conocarpus spp.) and neem trees. Extracts were prepared using maceration method with sequential polarization solvents including water (W), ethanol (E) and acetone (A) at different temperatures (35, 40 and 45°C) from leaves and fruits of C. lancifolius (CL), C. erectus (CE) and neem (N).

Aqueous extraction of C. erectus leaf at 40°C had the maximum content of phenolic components (1128.8±3.75 μg GAE/g). Furthermore, C. lancifolius and C. erectus fruit extracted with ethanol and acetone at 40° and 45°C, respectively, contained the maximum content of flavonoids (149.95±1.0 and 149.90±1.10 μg Catechol/g). While C. lancifolius leaf and fruit extracts, extracted by water and ethanol at 35°C, possessed the highest antioxidant activity (93.52±0.92% and 93.49±0.39 %, respectively).

In term of antibacterial effects, ethanol extract of CLF at 35°C had the highest effect on E. coli and P. aeruginosa. While aqueous extracts of CEL and CLL at 40 and 45°C respectively, and ethanolic extract of CEF at 45°C, had the greatest effects on Salmonella senftenberg. Furthermore, the highest inhibition zone on Yersinia enterocolitica is shown by CEL (extracted at 40°C with water) and CLF (extracted at 40°C with ethanol), while the highest inhibition for Campylobacter jejuni was obtained with ethanolic CLF at 40°C.

Regarding anti-weeds applications, CLF-45W and CLF-45E extracts had the highest inhibitory effects on bermudagrass seed germination (32.66% and 29.66%, respectively), which will help to suppress weed growth. Additionally, Conocarpus spp. was used to extract high concentration of gallic acid (7480 mg/kg), p-hydroxy benzoic acid (20217.23 mg/kg), chlorogenic acid (11522 mg/kg) and vanillic acid (14809.23 mg/kg), which can be employed in the pharmaceutical and food industries. Regarding food application as a fig fruit preservative, CEF-45E shows the lowest percentage of weight loss, while CLF-35E shows the best appearance and color.

### O15 Assessing the anthropometrics of young athletes in the United Arab Emirates

#### Seham Al Raish^1,^ Carine Platat^2^

##### ^1^Hemaya Institute for Health, Safety, Environment and Food Science, Sharjah Research; Technology and Innovation Park, Sharjah 66636, United Arab Emirates; ^2^Department of Nutrition and Health, College of Medicine and Health Sciences, United Arab; Emirates University, Al Ain 15551, United Arab Emirates

###### **Correspondence:** Seham Al Raish (200440261@uaeu.ac.ae)

*BMC Proceedings 2023*, **17(16):**O15

**Background**

Nutrition is an essential component of human health and development. However, the prevalence of overweight and obesity is increasing worldwide, as are obesity-related diseases. The skinfold measurement method is the most widely used body fat composition testing method for assessing body fat percentage. Body mass index (BMI) is an index of weight-for-height that is commonly used to classify weight category.

**Materials and methods**

The goal of this research was to determine the prevalence of body weight, body fat, and waist circumference. Body Mass Index (BMI) and body fat percentages were calculated by different skinfold thickness and by body fat analyzer and waist circumference were calculated for each subject, and WHO classification was used to define the cut points in a cross-sectional study among 59 male soccer players aged 13-18 years recruited from Al Jazira Academic sports clubs in the United Arab Emirates.

**Results**

According to the findings, the prevalence of underweight, overweight, and obesity was 1.69%, 6.7%, and 0%, respectively, with a healthy weight of 91.50%. The respondents' average mean body fat percentage as measured by a body fat analyzer was 16.463.28%. Biceps 4.662.20 mm, Triceps 7.442.58 mm, supra-iliac 7.552.94 mm, and subscapular 8.172.00 mm were the mean body fat percentages calculated by skinfold thickness. The average skinfold reading for athletes was 55.91% for triceps and 72.85% for sub-scapular. According to NCHS waist percentiles, 69.60% of athletes fell into the 5th - 25th percentile, 26.80% in the 50^th^, and the rest were in the 5^th^.

**Conclusions**

Our findings could be used in obesity awareness promotion and nutrition education programs because they show that some athletes have unhealthy weight, skin fold, and waist circumference when compared to others. However, more research into the determinants of obesity and body fat, such as age, gender, race, nutrition, and changes over time, is required.

**Acknowledgements**

We would to thanks both UAEU and Dr Amjed for their support.

### O16 Medicinal plants of United Arab Emirates: their database, traditional uses and natural source for drug discovery

#### Mohamed T Mousa

##### National Herbarium Supervisor, Biology Department, College of Science, UAEU, UAE

###### **Correspondence:** Mohamed T Mousa (mohamed.mousa@uaeu.ac.ae)

*BMC Proceedings 2023*, **17(16):**O16

This study aims at assimilating and screening the medicinal uses of endemic natural plants of the United Arab Emirates. More than 20 percent of United Arab Emirates fora were found to possess medicinal properties in the UAE traditionally, they differ from herbs, grasses, shrubs and trees. Asteraceae and Fabaceae families have the maximum number of species. Maximum number of medicinal plant species were recorded from mountains and wadi habitat.

Medicinal plants database showed that plants have different uses and can be used for diseases treatment. Acacia tortilis for infections, jaundice stomach acidity, Aerva javanica is used for stopping bleeding, anthelmintic, antibacterial, Capparis cartilaginea for childbirth, earache, kidney, Purgative problems, headache, bruises, paralysis, swellings and snakebite, Citrullus colocynthis used for diabetes and hypoglycemia, Heliotropium bacciferum for scorpion bites and snake bites, Leptadenia pyrotechnica as antibacterial diuretic and for Insect biting, Moringa peregrina for constipation, stomach cramp and for swellings, Nerium oleander coughs and bronchitis, Prosopis cineraria used as eye drops and bark used for rheumatism and applied to scorpion bites, Rhazya stricta used to improving bad breath, chest pain, fevers skin rash conjunctivitis, constipation and for diabetes, Salvadora persica applied for oral disease and for scorpion sting and skin blisters, Senna italica used to treat stomach cramps and for constipation and Tribulus terrestris Used for sexual function in humans and improve athletic performance

Medicinal plants database will be available online under UAEU-National Herbarium (UAEU-NH), for all informations , plants description and location

### O17 Time-dose response modeling of toxicants

#### Hussam Alrabaiah

##### College of Engineering, Al Ain University, UAE

###### **Correspondence:** Hussam Alrabaiah (hussam.alrabaiah@aau.ac.ae)

*BMC Proceedings 2023*, **17(16):**O17

Background

It is important to measure the degree of toxicant effect on biota. Most studies conducted in this regard assess mostly the concentration (or dose) of toxicants. In this study, a new factor is incorporated into the assessment procedure. Exposure time is an important factor in the assessment of their effect predictions.

Materials and methods

A two-dimensional time-concentration-response mathematical model has been developed with the guide of other modeling attempts on research papers conducted by others. The model was calibrated and validated from data and observations found in many studies in the literature. This is done by drawing different scenarios and different values of the parameters of the mathematical model.

Results and Conclusions

The model has proven its usefulness to predict the responses of organisms exposed to different levels of toxicants at different exposure durations. The model has been successfully validated in several sets of data available in the literature. The proposed model is a useful tool to predict population dynamics or survival analysis in toxicology experiments, particularly in aquatic environments, pharmacology studies, or Pharmacokinetics and hence to derive policy decisions concerning risk assessment in ecology or clinical pharmacy.

### O18 Soluble expression of halophilic enzymes: challenges and potential solutions

#### Nayla Munawar

##### United Arab Emirates University, Al-Ain, UAE

###### **Correspondence:** Nayla Munawar (nmunawar@uaeu.ac.ae)

*BMC Proceedings 2023*, **17(16):**O18

The use of enzymes in the industrial field has gained enormous attention as an alternative to classic chemical catalysis after global aspiration for sustainable industrial processes. Extreme environments offer microorganisms containing novel robust enzymes for biotechnological applications. Enzymes from halophilic (salt-loving) archaea especially have distinctive structural adaptations to maintain their integrity and catalytic efficiency in the high concentration of KCl, which cells accumulate to cope with their external osmotic pressure. Since salinity increases hydrophobic interactions and decreases the strength of ionic bonds, enzymes from halophilic archaea are naturally adapted to work under low water environments, hence are expected to work in non-polar solvents. Moreover, modern genetic and biochemical analytical tools have provided an opportunity to explore the structure and function of the cellular machinery of halophiles, revealing the enormous biotechnological potential of novel biocatalysts from these microbes. Halophilic enzymes are generally heat-resistant and organic solvent tolerant which makes them strong candidates for protein engineering to provide unique industrial biocatalysts. However, the major obstacle to utilizing halophilic enzymes as industrial catalysts is the insoluble expression of recombinant halophilic enzymes in mesophilic hosts like E.coli because of low salt concentration in these cells. Two potential strategies, to get the soluble and active expression of recombinant halophilic enzymes, and the advantages and disadvantages of these methodologies will be presented using the enzyme glutamate dehydrogenase from an extremely halophilic microorganism Halobacterium salinarum and fructosyltransferase enzymes from Halakalicoccus jeotgali B3T and Haloarcula marismortui as models.

### O19 Herbal medicines in the Middle East: A cross-sectional survey of community pharmacist’s perspective and knowledge

#### Banaz Jalil^1^, Abdallah Y Naser^2^, Fatima B Jeragh-Alhaddad^3,^, Faris El-Dahiyat^4,5^, Abdulrahman E Koshak^6^, Michael Heinrich^1^

##### ^1^Pharmacognosy and Phytotherapy, UCL School of Pharmacy, London, United Kingdom; ^2^Department of Applied Pharmaceutical Sciences and Clinical Pharmacy, Faculty of Pharmacy, Isra University, Amman, Jordan; ^3^Department of Pharmacy Practice, Faculty of Pharmacy, Kuwait University, Kuwait City, Kuwait; ^4^Clinical Pharmacy Program, College of Pharmacy, Al Ain, Al Ain, United Arab Emirates; ^5^AAU Health and Biomedical Research Center, Al Ain University, Abu Dhabi, United Arab Emirates; ^6^Department of Natural Products and Alternative Medicine, Faculty of Pharmacy, King Abdulaziz University 80260, Jeddah, 21589, Saudi Arabia

###### **Correspondence:** Banaz Jalil (b.jalil@ucl.ac.uk), Michael Heinrich (m.heinrich@ucl.ac.uk)

*BMC Proceedings 2023*, **17(16):**O19

Background

In the Middle East, many herbal medicines (HMs) are available on the markets, both those prepared locally or imported from other countries [1]. The discrepancies in regulatory status [1] and less stringent supply chains result in adulterated (herbal and other medical) products being sold with undeclared synthetic substances. This poses severe health risks to the public consuming these products. Our study evaluated the community pharmacist’s perception, recommendation and knowledge of the use and safety of HMs in three Middle Eastern countries (Kuwait, Saudi Arabia and the United Arab Emirates).

Methods

Between November 2021 and June 2022, a cross-sectional online survey was conducted using social media platforms. The study population included only community pharmacists working in the countries of research. The survey tool was developed and validated in previously conducted studies in the Middle East [2, 3].

Results

After ethical approval, a total of 255 responses (UAE n=108; Kuwait n=83; Saudi Arabia n=64) were obtained. Overall, HMs are sold in nearly all pharmacies (92.2%), with 76.7% reporting that their customers request HMs. 57.4% of respondents were aware of potential herb-drug interactions, 46.9% did receive complaints from customers about HMs, and 70.2% would report adverse reactions to the national pharmacovigilance services. The mean knowledge score was 13.6 [2.2] out of 20, which is equal to 68.0% out of the maximum attainable score.

Conclusions

This is one of the first studies exploring pharmacists’ perceptions and knowledge of HMs in the Middle East. Utilising the same validated study tools, comparisons can be made easily. This builds a foundation for a better understanding of the needs of community pharmacists in the countries of research. There are some limitations; with the small sample size, we could not estimate the response rate (non-response bias). Further studies are needed to enable a more robust assessment and determine whether these findings are transferable to other (Middle Eastern) countries.

References

1. World Health Organisation. WHO global report on traditional and complementary medicine 2019. World Health Organization; 2019. Report No.: 9241515430.
2. Jalil B, Naser AY, Prieto JM, Heinrich M. Herbal supplements in Jordan: a cross-sectional survey of pharmacists’ perspectives and knowledge. BMJ open. 2022;12(7):e057405.
3. Jalil BT. Herbal Supplements In The Middle East (Iraq And Jordan): Regulation, Quality And Safety Of And Development Of A Method To Detect Common Adulterants: UCL (University College London); 2021.

### O20 Higher education institutions’ contribution to SDG 3 achievement through pharmacy education: current practices and future possibilities in the UAE

#### Sumaya Daoud

##### Al Ain University, Al Ain, United Arab Emirates

###### **Correspondence:** Sumaya Daoud (sumaya.daoud@aau.ac.ae)

*BMC Proceedings 2023*, **17(16):**O20

Introduction

In September 2015, the UN set 17 Sustainability Development Goals (SDGs) to be achieved by 2030 to achieve a more sustainable future for all. To cope with the global efforts, the United Arab Emirates is striving through its organizations and institutions to work towards achieving these goals. Specifically, higher education institutions are more involved than ever in the national efforts towards SDGs achievement through three primary missions assigned to them; namely, teaching, research and community engagement. The present paper gives an account of current practices and strategies that are adopted by educators and researchers involved in pharmacy education at one of the UAE universities to achieve SDG 3 Good Health and Well-being. The paper attempts to offer proposals for future plans to enhance and accelerate the progress of the implementation of the sustainable development agenda in academia through pharmacy education.

Procedure

The researcher collects data from the college of pharmacy on the current procedures, practice and policies that are adopted to ensure the that SDG 3 has been addressed. For example, the courses will be examined in terms of their description and learning outcomes to find the extent to which SDG 3 is taken in to consideration their content. In addition, research and scholarly work will be discussed in terms of solutions they offer to actual health problems that are being faced locally or globally. Community engagement activities will be discussed as to whether they have an impact on the community in raising awareness and promoting good health for all. Finally, future directions will be addressed based on evidence from success stories from other higher education institutions working towards the same goal.

### O21 Phytochemical analysis and in vitro thrombolytic activity of Embelia robusta and Dracaena reflexa

#### Saad Touqeer^1^, Umair Ikram Dar^2,3^, Salman Hamid^2^, Farooq Saleem^2^, Muhammad Asad Saeed^2^

##### ^1^Al Ain University AD campus, UAE; ^2^Department of Pharmacy, The University of Lahore, Lahore, Pakistan; ^3^College of Pharmaceutical Sciences, The University of Lahore, Lahore, Pakistan

###### **Correspondence:** Saad Touqeer (saad.touqeer@aau.ac.ae)

*BMC Proceedings 2023*, **17(16):**O21

Thrombosis is a condition in which the normal haemostatic processes are disturbed resulting in the formation of clots in the uninjured blood vessels, which may cause partial or complete occlusion of that vessel. The thrombus formed in any blood vessel may either propagate and obstruct the vessel completely or it may be dislodged and move to any secondary site (thromboembolism). Both of the conditions can be fatal as loss of blood supply may lead to infarction of vital organs of the body. The organs mostly affected by thrombosis include heart, brain, spleen and kidney [1]. The currently available thrombolytic therapy has several drawbacks. Serious adverse effects associated with thrombolytic drugs, together with their high cost make it necessary to search for new drugs. Natural products may serve as a good source for new drugs due to their better safety profile [2-4]. In this context, we carried out an in-vitro clot dissolution assay on the methanolic extracts of the whole plant of Dracaena reflexa and the fruit of Embelia robusta [5]. The percentage of clot lysis was determined at a dose of 200, 400 and 800 (μg/ml) at intervals of 1.5, 24, 48 and 72 hours. The results showed both extracts to have significant thrombolytic activity (p < 0.05) at doses of 400 and 800 (μg/ml) in both acute and choric studies and were found to be comparable to the standard drug streptokinase. Preliminary phytochemical studies also revealed the presence of major classes of secondary metabolites such as alkaloids, glycosides, flavonoids and tannins. Saponins were present only in D. reflexa methanolic extract. The study confirms the potential of the two plants as potent thrombolytic agents.

References

1. Furie B, Furie B.C. Mechanisms of thrombus formation. New England Journal of Medicine. 2008: 359; 938-949.
2. Betancourt BY, López G, Silva C, Iglesias E, Bernal F, Saura PA. Pharmacovigilance program to monitor adverse reactions of recombinant streptokinase in acute myocardial infarction. BMC Pharmacology and Toxicology. 2005: 5; 5-10.
3. Malik J A, Khan G Q. Adverse effect profile of streptokinase therapy in patients with acute myocardial infarction: a prospective study. JK practitioner. 2004: 11; 106-109.
4. Sherwani S K, Khan M M, Zubair A, Shah M A, Kazmi S U. Evaluation of In Vitro Thrombolytic Activity of Bougainvillea Spectabilis Leaf Extract. Int. J. Pharm. Sci. Rev. Res. 2013: 21; 6-9.
5. Mowla TE, Zahan S, Sami SA, Uddin SN, Rahman M. Potential effects and relevant lead compounds of Vigna mungo (L.) Hepper seeds against bacterial infection, helminthiasis, thrombosis and neuropharmacological disorders. Saudi Journal of Biological Sciences. 2022: 29;3791-805.

### O22 Design and development of phosphoinositide-3-kinase (PI3Kα) inhibitors

#### Dima A Sabbah

##### Al-Zaytoonah University of Jordan, Jordan

###### **Correspondence:** (dima.sabbah@zuj.edu.jo)

*BMC Proceedings 2023*, **17(16):**O22

Background

The phosphatidylinositol 3-kinase (PI3Kα) has been recognized as a significant oncogene and therapeutic target for anticancer drug design.

Objective

Target compounds were designed recruiting ligand- and structure-based drug design strategies to probe the influence of the compounds’ core structures and binding groups on the biological activity.

Methods

Synthesis of the targeted compounds, biological examinations against human cancer cell lines, and molecular docking studies.

Results

Fortunately, 20 novel series of diverse nuclei were synthesized and characterized using FT-IR, 1H and 13C NMR, HRMS, and elemental analysis. In addition, the identity of one backbone was successfully characterized by X-ray crystallography. Biological activity of synthesized compounds was screened in vitro against human cancer cell lines. Results showed that the analogues exert antiproliferative activity and incite apoptosis by increasing caspase-3 activity and decreasing DNA cellular composition. In addition, ligand-based pharmacophore modeling delineated that the recently synthesized scaffolds approve PI3Kα inhibitors pharmacophore and the molecular docking approaches against PI3Kα depicted that the analogues accommodate PI3Kα kinase domain and engage with key binding residues.

Conclusion

The derivatives displayed a possible PI3Kα suppression activity in human cancer cell lines.

Future Goal: Screening the prospective analogues against a kinase array to retrieve selective inhibitors.

### O23 The impact of augmented reality applications on Emirati grade 12 students' achievement and attitudes towards health science education

#### Saif Saeed Salem Al Neyadi

##### Al Ain University, Al Ain, United Arab Emirates

###### **Correspondence:** Saif Saeed Salem Al Neyadi (saif.alneyadi@aau.ac.ae)

*BMC Proceedings 2023*, **17(16):**O23

The study attempted to explore the effectiveness of using augmented reality applications in deepening Grade 12 students' understanding health science learning. Additionally, it aimed at investigating the impact of augmented reality applications on grade 12 students’ performance as measured by cognitive outcomes and attitudes. This aim could be achieved by comparing the results of two groups; the experimental group learnt a unit of understanding cancer cells shape and growing in the human body by of augmented reality applications-based instruction while the control group learnt the unit in the traditional lecturing instruction. Three research questions are used to guide the study. A mixed-method approach was utilized in conjunction with an exploratory sequential design to explore the impact of augmented reality applications on learners' performance and attitudes in the health sciences subject, where students' performance involves applying the technique. The results showed that the students' performance in applying ranked the highest (Mean =4.1), followed by knowledge (Mean = 3.9) while reasoning was the lowest (Mean =3.5). Grade 12 students learning via augmented reality applications performed higher than their counterpart students learning via traditional methods in all indicators: knowledge, applying and reasoning. The results of the qualitative data showed that most students developed positive attitudes towards the factors of scientific inquiry, enjoyment, and career interest for augmented reality applications by roughly 30%, 35%, and 35%, respectively. Upon the results, it is recommended to employ virtual environment in teaching and learning health science in terms of quality and quantity. Schools also needs to seize the progression of digital technology to achieve full integration to enhance teaching, learning as well as attitudes.

Keywords

Application subject, augmented reality -based instruction, lecture-based instruction, unit of cancer cells, science performance. science attitudes.

### O24 Exploring pharmacists’ perceptions towards telepharmacy implementation after COVID-19 pandemic

#### Mohamed Baraka, Asim Alnour, Adel Sadeq

##### Clinical Pharmacy Department College of Pharmacy Al Ain University Al Ain, Abu Dhabi, UAE

###### **Correspondence:** Mohamed Baraka (mohamed.baraka@aau.ac.ae)

*BMC Proceedings 2023*, **17(16):**O24

Background

Telepharmacy is defined as the action of provision of the pharmaceutical care services using video and audio tools. It provides the same traditional pharmaceutical care services using telecommunication tools. Patients suffering from chronic or infectious diseases and elderly are the patients expected to benefit the most from telepharmacy services. For patients to benefit from these services, they just need a microphone, camera, and a stable internet connection, and that simple equipment are readily available in many smart devices these days. Due to the quarantine applied by governments during the pandemic, patients may have had limited access to pharmacies and hospitals to obtain the needed pharmaceutical care, so the need for implementing such service in UAE after the pandemic is currently in high-demand. Objective: This project intended to explore pharmacists’ perceptions and preparedness towards telepharmacy implementation after COVID-19 pandemic. Moreover, to identify facilitators and challenges towards implementing such services. Materials & Methods: a survey-based cross sectional was designed to collect data from UAE registered pharmacists to explore their perceptions and preparedness towards implementation of telepharmacy after COVID-19 pandemic. As observational study, it was exempted by the ethics committee from ethical approval. Result: The study findings revealed that (75%) of pharmacists agreed that telepharmacy is expected to improve patients’ adherence. Similar percentage (75%) of pharmacists believe that comprehensive counseling can be done for patients via telepharmacy services. Around (56%) of pharmacists agreed that implementing telepharmacy will raise the level of job satisfaction. The vast majority of pharmacists (88%) identified training as one of the challenges towards implementing telepharmacy. Conclusion: Pharmacists believe that telepharmacy implementation is important for improving patients’ adherence, can help in remote patients counseling and enhancing their job satisfaction. However, they consider training as one of the major challenges towards the implementation of such services. Key words: Telepharmacy, Pharmacists, COVID-19 pandemic, Telehealth, Digital health.

### O25 The effect of methylation on autophagy in lung cancer cell lines

#### Rose Ghemrawi^1,2^, Aya Al Qassem^1,2^, Raghad Al-Dulaymi^1,2^, Azza Ramadan^1,2^

##### ^1^College of Pharmacy, Al Ain University, Abu Dhabi Campus, United Arab Emirates; ^2^AAU Health and Biomedical Research Center, Al Ain University, Abu Dhabi, United Arab Emirates

###### **Correspondence:** Rose Ghemrawi (rose.ghemrawi@aau.ac.ae)

*BMC Proceedings 2023*, **17(16):**O25

Background

The dualistic role of autophagy, in both the suppression and propagation of carcinogenesis, makes the link between autophagy and cancer far from being well understood. Moreover, innovative therapeutic strategies are needed for the treatment of cancer and especially lung cancer categorized as not curable because of the suboptimal treatment outcomes despite chemotherapy and immunotherapy. Recently, accumulating evidence has revealed that autophagy is regulated by proteins that are post-translationally modified, such as methylation. The goal of this study was to investigate the effects of the pharmacological inhibition of methylation on autophagy in lung cancer.

Methods

Experiments were performed in vitro on two lung cancer cell lines (H292 and A549) using the global methyltransferase inhibitor, Adenosine dialdehyde (AdOx). Cellular proliferation was assessed by MTT test, the migration by wound healing assay and the autophagy by evaluating the expression of autophagy markers (p62, ATG7, LCIII) through western blot.

Results

The inhibition of methyltransferase activity reduced lung cancer lines proliferation and wound closure. This was correlated with a decreased expression of p62 and an increased expression of ATG7 and LCIII, therefore an activation of autophagy.

Conclusion

We successfully found a relationship between methylation, autophagy and carcinogenesis. By unraveling the molecular mechanisms linking autophagy to tumorigenesis, our study opens novel therapeutic perspectives for the treatment of lung cancer.

### O26 Investigation of the interactions between probiotics and orally administered xenobiotics

#### Sara Mousa^1^, Dana Obaid^1^, Muhammad Sarfraz^1^, Walaa Mousa^1,2^

##### ^1^College of Pharmacy, Al Ain University, Abu Dhabi, UAE; ^2^College of Pharmacy, Mansoura University, Mansoura, Egypt

###### **Correspondence:** Sara Mousa (sara.mousa@aau.ac.ae)

*BMC Proceedings 2023*, **17(16):**O26

Background The use of probiotics as a beneficial supplement has been a common practice in the community. Although they are a supplement, the safety of co-administrating them with other xenobiotics remains undetermined. The gut microbiota has been recently recognized as a major manipulator of many orally administered xenobiotics. Therefore, it is very possible for probiotics to have a similar role since they contain bacterial strains present in the gut. The aim of this study was to investigate the interactions of probiotics and select orally administered xenobiotics from different drug classes.

Materials and Methods Aerobic bacterial strains, probiotics, and different classes of xenobiotics were purchased. Aerobic bacterial strains and probiotics were cultured. Based on the xenobiotic tested, assays were designed to accurately determine the effect of prepared cultures on the xenobiotics and vice versa. A computational search using NCBI’s database was performed to determine the spread of certain bacterial enzymes in the gut.

Results The activity of certain xenobiotics was altered when co-cultured with probiotics. Similarly, the growth of probiotics and aerobic bacterial strains was affected. It was either increased, decreased, or remained unchanged. The computational search demonstrated a widespread of bacterial metabolizing enzymes across gut microbes.

Conclusions These results illustrate the importance of establishing strict control over probiotics use. Along with additional measures to test the quality of produced probiotics.

References

1. Zimmermann M, Zimmermann-Kogadeeva M, Wegmann R, Goodman AL. Mapping human microbiome drug metabolism by gut bacteria and their genes. Nature. 2019;570(7762):462–7.
2. Ogbogu U, Necyk C. Community pharmacists’ views and practices regarding natural health products sold in community pharmacies. PLoS One. 2016;11(9):1–19.
3. Sanders ME, Akkermans LMA, Haller D, Hammerman C, Heimbach J, Hörmannsperger G, et al. Safety assessment of probiotics for human use. Gut Microbes. 2010;1(3):164–85.
4. Maier L, Pruteanu M, Kuhn M, Zeller G, Telzerow A, Anderson EE, et al. Extensive impact of non-antibiotic drugs on human gut bacteria. Nature [Internet]. 2018;555(7698):623–8. Available from: 10.1038/nature25979

### O27 The impact of a clinical pharmacist-managed anticoagulation clinic on patients’ outcomes and adherence

#### Meriam Alomari^1^, Faris El-Dahiyat^1,2^, Ammar Alhabib^3^, Ammar Jairouni^4^

##### ^1^Clinical Pharmacy Department, Al Ain University, Al Ain, UAE; ^2^AAU Health and Biomedical Research Center, Al Ain University, Abu Dhabi, United Arab Emirates; ^3^Clinical Pharmacy Department, Sheikh Shakhbout Medical City, Abu Dhabi, UAE; ^4^Health and Safety Department, Dubai Municipality, Dubai, UAE

###### **Correspondence:** Meriam Alomari (meriam6m@yahoo.co.uk)

*BMC Proceedings 2023*, **17(16):**O27

Background

For years, warfarin therapy has been the only oral anticoagulation drug used for the prevention of primary and secondary thromboembolic events [1]. With its narrow therapeutic index and related complications, the anticoagulation clinic (ACC) is one of the most needed services for warfarin therapy. The aim of the study is to show evidence of the importance of clinical pharmacist intervention in terms of improving adherence and managing patients using warfarin therapy in ACC.

Materials and Methods The research design is a pragmatic, prospective, quasi-experimental study used of patients attending ACC managed by the clinical pharmacist. The baseline data will be collected retrospectively from patients’ medical records from January to November 2021 when the patients who are eligible were attending the usual care (UC) clinics managed by physicians. The same patients followed up between December 2021 to October 2022 in ACC, the collected data prospectively will be compared at the end of the study to measure time in therapeutic range (TTR) by The Rosendaal method which is a recognized way to measure the effectiveness of warfarin therapy in a period of time [2]. In addition, adherence was assessed by using Morisky Medication Adherence Scale (MMAS-4) questionnaire before and after visiting ACC. The data were analyzed by using SPSS Software Version 24. using a 95% confidence interval of differences and a significance level of 0.05 is considered.

Results

There is a statistically significant increase in TTR after implementing ACC (P=0.023) compared to UC (70.25% VS 61.21%). Moreover, MMAS-4 scores were statistically significantly improved (P=0.001) compared to UC (0.32 VS 0.61).

Conclusion

In conclusion, providing this model of ACC managed by clinical pharmacists improved patients’ TTR and adherence to warfarin therapy.

Acknowledgment

A big thanks go to Sheikh Shakhbout Medical City (SSMC) and Al Ain University for supporting to completion of this research.

References

1. Ageno W, Gallus AS, Wittkowsky A, Crowther M, Hylek EM, Palareti G. Oral anticoagulant therapy - Antithrombotic therapy and prevention of thrombosis, 9th ed: American College of Chest Physicians evidence-based clinical practice guidelines. Chest. 2012;141(2 SUPPL.):e44S-e88S.
2. Rosendaal FR, Cannegieter SC, Van der Meer FJ, Briet E. A method to determine the optimal intensity of oral anticoagulant therapy. Thrombosis and haemostasis. 1993;69(03):236-9.
3. Phillips KW, Ansell J. Outpatient management of oral vitamin K antagonist therapy: Defining and measuring high-quality management. Vol. 6, Expert Review of Cardiovascular Therapy. 2008. p. 57–70.

### O28 Structure-informed antiviral drug discovery – A tale of two viruses

#### Mark von Itzstein

##### Institute for Glycomics, Griffith University, Gold Coast, Queensland 4222, Australia

###### **Correspondence:** Mark von Itzstein (m.vonitzstein@griffith.edu.au)

*BMC Proceedings 2023*, **17(16):**O28

Respiratory viruses can present the most challenging and life-threatening infections to humans and the current COVID-19 pandemic is testament to that potential. Influenza viruses through to coronaviruses continue to provide humanity with great concern due to the rapid onset of disease, as well as their potential overwhelming direct life-threatening impact on lung function and other organ systems. Furthermore, the rapid development of mutants may also lead to further complications in the employment of existing vaccines, where they exist. Consequently, antiviral drug discovery strategies to tackle respiratory viruses are of a high priority and in this lecture our engagement of structure-guided antiviral drug discovery will be presented. Using the structures of identified proteins that are critical in the virus’ lifecycle and targeting highly-conserved domains within these proteins, we have been able to develop potent inhibitors of influenza virus and human parainfluenza virus. Employing an integrated interdisciplinary approach that combines, structural and computational biology, chemistry and virology we have explored the active sites of neuraminidase and haemagglutinin- neuraminidase from influenza virus and human parainfluenza virus, respectively. Some of our unpublished preliminary data using fragment-based structure-guided drug discovery will also be presented.

### O29 Improving vaccination uptake – Using protection motivation theory to understand vaccine hesitancy

#### Jonathan Ling^1^, Walid Al-Qerem^2^, Judith Eberhardt^3^

##### ^1^Faculty of Health Sciences & Wellbeing, University of Sunderland, UK; ^2^Faculty of Pharmacy, Al Zaytoonah University of Jordan, Jordan; ^3^Department of Psychology, Teesside University, UK

###### **Correspondence:** Jonathan Ling (jonathan.ling@sunderland.ac.uk)

*BMC Proceedings 2023*, **17(16):**O29

COVID-19 booster vaccine uptake has been below the uptake of the first and second dose of the vaccine in many countries. Approaches to COVID-19 vaccination have varied between countries, with some (e.g., Germany, Austria) considering or temporarily implementing mandatory vaccination, whilst others (e.g., the United Kingdom, Jordan) have not. Protection Motivation Theory (PMT) has been applied to COVID-19 vaccination intention, but little is known about the role of PMT in booster vaccination intention, nor in relation to any differences between countries using different approaches to vaccination. Furthermore, whilst social media use and sociodemographic factors such as religiosity play a role in COVID-19 vaccination intention, their role in booster vaccination intention is unclear. This study aimed to predict COVID-19 booster vaccination intention using PMT, coronavirus conspiracy beliefs, social media use, and sociodemographic factors, and compare these between the United Kingdom, Jordan, Germany, and Austria. We found that while PMT constructs predict booster vaccination intention, additional factors such as conspiracy beliefs, social media use, and religiosity need to be taken into account in public health campaigns to increase COVID-19 booster dose uptake.

### O30 The experience of patients diagnosed with COVID-19 infection, and the role of the pharmacist during their infection: a case study from the United Arab Emirates

#### Iman A Basheti^1,2^, Hiba Barqawi^3, 4^, Razan I Nassar^5^, Samar Thiab^5^, Noor Atatreh^6,7^, Eman Abu-Gharbieh^8,9^

##### ^1^Department of Clinical Pharmacy and Therapeutics, Faculty of Pharmacy, Applied Science Private University, Amman 11931, Jordan; ^2^Faculty of Medicine and Health, School of Pharmacy, The University of Sydney, Camperdown, NSW 2006, Australia; ^3^Research Institute of Medical and Health Sciences, University of Sharjah, Sharjah 27272, United Arab Emirates; ^4^Department of Clinical Sciences, College of Medicine, University of Sharjah, Sharjah 27272, United Arab Emirates; ^5^Department of Pharmaceutical Chemistry and Pharmacognosy, Faculty of Pharmacy, Applied Science Private University, Amman 11931, Jordan; ^6^College of Pharmacy, Al Ain University, Abu Dhabi 64141, United Arab Emirates; ^7^AAU Health and Biomedical Research Center, Al Ain University, Abu Dhabi 64141, United Arab Emirates; ^8^Research Institute of Medical and Health Sciences, University of Sharjah, Sharjah 27272, United Arab Emirates; ^9^Department of Clinical Sciences, College of Medicine, University of Sharjah, Sharjah 27272, United Arab Emirates

###### **Correspondence:** Iman A Basheti (dr_iman@asu.edu.jo)

*BMC Proceedings 2023*, **17(16):**O30

Recognizing patients' experiences can produce encouraging results in the treatment of coronaviruses. Pharmacists play a crucial role in managing patients' experiences during their COVID-19 infection. The healthcare industry's needs have changed due to the new experiences that coronavirus patients have. In managing coronaviruses, acknowledging patients' experiences can show promising results. Pharmacists are key in managing patients' experiences while they are ill. Assessing the experience of COVID-19 infected people in the United Arab Emirates and the role of pharmacists during their infection were the main objectives. A cross-sectional study was carried out in June and July 2022. After an extensive literature review, the survey's face and content were validated. The survey was divided into three sections (demographics, experience of infected individuals, and role of pharmacists). The Statistical Package for the Social Sciences was used to analyze the data. The study's 509 participants had an average age of 34.50 (standard deviation: 11.93). About three-quarters of the participants had already received a vaccination, and more than half had at least one infection. Among the participants, fatigue (81.5%), fever (76.8%), headache (76.6%), dry cough (74.1%), muscle or joint pain (70.7%), and sore throat (68.6%) were the symptoms most frequently reported. The most popular dietary supplement was vitamin C (88.6%), followed by analgesics (78.2%). The only variable linked to the severity of symptoms was the female gender. 79.3% of participants exposed to COVID-19 strongly agreed or agreed that the pharmacist had played a crucial and successful role during their infection. Females reported more severe symptoms than males, with fatigue, fever, headaches, dry coughs, and muscle or joint pain the most frequently reported symptoms. During this pandemic, the pharmacist's role was crucial, particularly when advising patients on how to manage their viral infection and how to use their medications while infected.

### O31 Assessment of the clinical pharmacist's knowledge, skills, and competencies in Evidence-Based Medicine: an interrupted time series design

#### Sahar M Mohamed^1^, Asim A Elnour^2,3^, Khalid Ghalib^4^, Salma M Alhaj^5^, Fariha Mostafiz^6^

##### ^1^College of Pharmacy, University of Khartoum, Khartoum-Sudan; ^2^Program of Clinical Pharmacy, College of Pharmacy, Al Ain University, Abu Dhabi campus, Abu Dhabi-United Arab Emirates; ^3^AAU Health and Biomedical Research Center, Al Ain University, Abu Dhabi, United Arab Emirates; ^4^Consultant physician - Ibrahim Malik Hospital, Khartoum-Sudan. Assistant Professor of Medicine - Nahda College - Khartoum-Sudan; ^5^Clinical Pharmacist, College of Pharmacy, University of Khartoum. Khartoum-Sudan. Clinical pharmacist, Fedail hospital, Khartoum-Sudan; ^6^College of Pharmacy, Al Ain University, Abu Dhabi Campus, Abu Dhabi-United Arab Emirates

###### **Correspondence:** Asim A Elnour (asim.ahmed@aau.ac.ae)

*BMC Proceedings 2023*, **17(16):**O31

Background

Evidence-Based Medicine [EBM] is important area for educational research. The utilization of EBM by the clinical pharmacist is a growing field; however, very scarce research conducted in this respect.

Objective

The aim of the current research is to assess the impact of educational (Fresno's test of competency) and behavioral interventions on improving the skill and competencies of clinical pharmacist in EBM.

Materials and methods

The study conducted in Khartoum state hospitals, Sudan, where eligible clinical pharmacists’ recruited, joined the delivered training program, and asked to complete a pre and post-online test. The educational program presented via Google Meet, assessed, and evaluated online, in addition to and WhatsApp group for follow up and sharing of materials. We used the validated Frenso’s test, and the GREET checklist to report the educational process of EBM. The educational program consisted of total 38 hours for one month and half. 20 hours lectures online via Google Meet, 6 hours of continuous assessments, and 12 hours self-directed learning by sharing references in WhatsApp group. The ratio of instructors to clinical pharmacists was 4:77 (1 main instructor, 3 assistants).

Results

The finding showed higher mean scores in posttest, revealing the true impact of educational intervention. The results confirmed that the clinical pharmacist’s performance (knowledge, skills, and competences) in EBM improved upon in posttest. The statistical output indicates that the mean for pretest scores was 20.04, and for posttest scores was 70.95. The average difference between the paired pretest and posttest scores -50.9 in favor of the posttest (CI posttest -50.010 to 43.808, P < 0.001).

Conclusion

Our findings provided evidence for the usefulness of Frenso’s test assessment to improve the performance of clinical pharmacists’ uptake of EBM principles for future implementation into their respective practices.

## Poster Presentations

### P1 The awareness and barriers towered breast cancer screening

#### Mohammad A Al-Ghazali, Abdulkarim M Alshammakhi, Fatma M Algabri, Sondus S Almamari

##### Department of Pharmacology and Biology, College of Pharmacy, National University for Science & Technology, Muscat, Oman

###### **Correspondence:** Mohammad A Al-Ghazali (mohammadalghazali@nu.edu.om)

*BMC Proceedings 2023*, **17(16):**P1

Background

Breast cancer mostly affects women and represents the most common type of cancer worldwide. The prevalence has still increased even though the new process for treatment and diagnosis has developed [1]. The expected increase in cancer in 2040 is fifty percent higher than 2020 [2]. Breast cancer is the most common cancer among women in Arab countries with a young age of around 50 years [3]. In Oman, breast cancer was classified as the most common cancer type among females [4].

Purpose

This study aimed to evaluate the awareness and barriers of women toward breast cancer screening.

Methods

This is a pilot study which was conducted among women in Oman and includes females who didn’t have a history of breast cancer. It was conducted among students and staff in several hospitals. A total of 207 responses were received from 250 participants.

Results

The response rate in this study is 82.8% and most of the participants were within the age of 20-40 years (64%). It was noticed that although most women (92%) thought that breast cancer could be cured if detected early, more than 40% do not know whether breast cancer screening is effective or not. Moreover, 44 % will get their first screening mammogram only when they suspect a lump formation and 68% were aware that breast cancer screening was available in Oman and free of charge. However, the majority agreed that social barriers and embarrassment (73%) are the major reasons that may lead to delay in seeking medical help.

Conclusions

Awareness of Brest caner is higher in this study in comparison with previous studies. Although women showed an interest in breast cancer screening, several cultural, practical, and personal-related barriers were noted to interfere with breast cancer screening.

Keywords

Breast cancer, Awareness, Barriers, Screening

### P2 Monoacylglycerol lipase inhibitors relieve chemotherapy-induced neuropathic pain: Studies in mice models

#### Willias Masocha, Altaf Al-Romaiyan

##### Department of Pharmacology and Therapeutics, Faculty of Pharmacy, Kuwait University, Safat, Kuwait

###### **Correspondence:** Willias Masocha (willias.masocha@ku.edu.kw)

*BMC Proceedings 2023*, **17(16):**P2

Background

The use of paclitaxel against cancer is limited by development of chemotherapy-induced neuropathic pain (CINP). The endocannabinoid 2-arachidonoyl glycerol (2-AG) has antinociceptive activity, however it is rapidly metabolised by the enzyme monoacylglycerol lipase (MAGL). Thus, the objective of this study was to evaluate whether MAGL inhibitors could prevent development of or treat CINP in an animal model.

Methods

Paclitaxel was intraperitoneally administered to female BALB/c mice to induce mechanical allodynia. LC-MS/MS was used to measure 2-AG, PCR to measure Mgll gene transcripts, Wes™ to measure MAGL protein expression and assay kits to measure MAGL activity. The effects of pristimerin on the activity human recombinant MAGL (hrMAGL) and mouse MAGL (mMAGL) activity in tissues were evaluated using MAGL assay and inhibitor screening kits. The effects of treatment of mice with pristimerin intraperitoneally and 2-AG and JZL184 (MAGL inhibitor) subcutaneously into the right hind paw on paclitaxel-induced mechanical allodynia was measured using the dynamic plantar aesthesiometer.

Results

Mice treated with paclitaxel developed mechanical allodynia, no change in Mgll transcripts or mMAGL protein expression but had increased mMAGL activity and had reduced levels of 2-AG in the paw skin. Pristimerin inhibited hrMAGL activity and mMAGL activity in the paw skin. Treatment with pristimerin inhibited the paclitaxel-induced increase in mMAGL activity. Pristimerin prevented development of paclitaxel-induced mechanical allodynia. 2-AG and JZL-184 produced localised antiallodynic effects in the injected paw. The antiallodynic effects of 2-AG were blocked by both cannabinoid type 1 (CB1) and CB2 receptors inhibitors AM251 and AM630, respectively.

Conclusion

During paclitaxel-induced mechanical allodynia there is an increase in mMAGL activity, and a deficiency of 2-AG. Co-treatment with MAGL inhibitors and paclitaxel could be useful in the treatment and prevention of CINP.

Acknowledgements

This work was supported by grant PT01/20 and RCF grants SRUL02/13, GM01/13 from Kuwait University Research Sector.

### P3 Impact of processing and preservation methods on total phenolics, flavonoids and antioxidant activities of Okra (Abelmoschus esculentus L.)

#### Maher Al-Dabbas^1^, Majd Mumneh^2^, Mahmoud Abu-Ghoush^1^, Balkees Abuawad^1^

##### ^1^College of Pharmacy- Department of Nutrition and Dietetics- Al Ain University- Abu Dhabi, UAE; ^2^The University of Jordan, Jordan

###### **Correspondence:** Maher Al-Dabbas (maher.dabbas@aau.ac.ae)

*BMC Proceedings 2023*, **17(16):**P3

The present study was undertaken to investigate the influence of blanching, freezing, frying, sun drying and dehydration conditions on okra total phenolic, total flavonoids and antioxidant activities after processing and during storage. Fresh okra was dried using sun drying and conventional oven drying at 70 C. Blanching, frying and freezing were accomplished according to the traditional methods of preservation. Ethanolic extract of each sample were analyzed before and after preservation every month for a period of three months. The results showed a significant improvement (p < 0.05) in the total phenolic (134.1 mg GAE/ 100g) and DPPH (1-1- diphenyl1-2-pricrylhydrazyland) scavenging activity (IC50 value of 3.0 mg/ml) in the blanched okra compared with fresh okra (86.35 mg GAE/ 100g and 3.8 mg/ml, respectively). Fresh okra showed the highest flavonoids contents (105.75 mg QE/100g), whereas sun dried okra and after 3 months of storage showed to contain the lowest total phenolic (14.450 mg GAE/ 100g), total flavonoid contents (13.250 mg QE/100g), reducing power activity (23.30%) and the lowest DPPH scavenging activity (IC50 value of 134.8 mg/ml). The DPPH inhibition activities of all treatments of okra showed strong correlation with the okra phenolic contents and flavonoids content (r =0.702 and 0.67, respectively). The reducing power activity (%) of all okra treatments exhibited strong correlation with phenolic contents (r = 0.966), while with flavonoid contents the correlation (r) was = 0.459. In general, different preservation methods of okra resulted in decreasing of the total phenolic content and freezing shown to be the highest in retention of total phenolic and flavonoids contents and antioxidant activities, while sun dried okra was the lowest in their retention.

### P4 Methods of assessment of polysialyltransferase inhibitors for treatment of tumour cell dissemination

#### Xiaoxiao Guo^1^, Jodie R Malcolm^2^, Anjana Patel^1^, Marrwa M Ali^1^, Goreti R Morais^1^, Steven D Shnyder^1^, Paul M Loadman^1^, Laurence H Patterson^1^, Robert A Falconer^1^

##### ^1^Institute of Cancer Therapeutics, Faculty of Life Sciences, University of Bradford, Bradford BD7 1DP, United Kingdom; ^2^York University, United Kingdom

###### **Correspondence:** Robert A Falconer (r.a.falconer1@bradford.ac.uk)

*BMC Proceedings 2023*, **17(16):**P4

Polysialic acid decorates the surface of NCAM on neuroendocrine tumours, notably neuroblastoma and small cell lung carcinoma, and is strongly associated with poor prognosis and aggressive disease in patients in the clinic. PolySia modulates tumour cell-cell and cell-matrix adhesion, migration, invasion and metastasis. SiRNA knockdown of polysialyltransferase (polyST) ST8SiaII, the enzyme primarily responsible for polySia synthesis in tumours, abrogates tumor cell migration and invasion. PolyST is a selective and largely unexplored therapeutic target for neuroblastoma dissemination. While assays are available to assess polyST enzyme activity, there is no methodology available specifically optimized for identification of novel polyST inhibitors. We describe the development of cell-free and cell-based assays that enable assessment of polysialyltransferase inhibition.

Development of the HPLC-fluorescence-based enzyme assay includes a comprehensive optimization of assay conditions, including evaluation of metal ion composition, enzyme concentration, substrate and acceptor concentration, temperature, pH and tolerance to DMSO, followed by validation using known polyST inhibitors. Under these optimized conditions, the experimentally observed Ki value for CMP, a competitive polyST inhibitor, was strongly correlated with the predicted Ki value, based on the classical Cheng-Prusoff equation [average fold error (AFE) = 1.043]. These results indicate that this assay can provide medium-throughput analysis for enzyme inhibitors with high accuracy. We additionally report optimized HPLC-based and ELISA-based methodologies for assessment of polyST inhibition in neuroblastoma cells, using endoneuraminidase N as control, and assessment of ICT3176 (a polysialylation inhibitor) as a test agent.

In conclusion, in vitro cell-free and cell-based assays for accurate measurement of polysialyltransferase inhibition are described, specifically designed for routine identification of potential polyST inhibitors, generation of kinetics data and assessment of mode of inhibition and effects on cellular polysialylation. Given the growing interest in the polySTs as important anti-metastatic target, these are vital tools to enable preclinical identification of novel polyST inhibitors for neuroblastoma therapy.

### P5 Food safety and quality assessment of the foodstuff served in the fast foods restaurants and their role on the consumer health and perception during COVID-19

#### Mahmoud Abughoush^1,2^, Amin N Olaimat^2^, Murad A Al-Holy^2^, Maher Al-Dabbas^1,3^, Sajid Alavi^4^,

Sofyan Maghaydah^5,6^, Imranul Choudhury^7^

##### ^1^Science of Nutrition and Dietetics program, College of Pharmacy, Al Ain University. P.O. Box 64141, Abu Dhabi, UAE; ^2^Department of Clinical Nutrition and Dietetics, Faculty of Applied Medical Sciences, The Hashemite University, P.O. Box 330127, Zarqa 13133, Jordan; ^3^Department of Nutrition and Food Technology, Faculty of Agriculture, University of Jordan, Jordan; ^4^Kansas State University, Manhattan, Kansas, USA; ^5^Department of Nutrition and Food Technology, Faculty of Agriculture, Jordan University of Science and Technology, P.O. Box 3030, Irbid 22110, Jordan; ^6^Department of Human Nutrition and Dietetics, College of Health Sciences, Abu Dhabi University, Zayed City, Abu Dhabi, United Arab Emirates; ^7^College of Pharmacy, Al Ain University. P.O. Box 64141, Abu Dhabi, UAE

###### **Correspondence:** Mahmoud Abughoush (mahmoud.abughoush@aau.ac.ae)

*BMC Proceedings 2023*, **17(16):**P5

The quality and the safety of the foods that are served at fast foods restaurants and their effect on the consumer health could become a matter of concern during COVID-19. No study has critically evaluated the quality, safety and the acceptability of the foods that are served at these fast foods restaurants in the different universities in Jordan during COVID-19. Therefore, our main goal in this study was to evaluate the effect of fast foods consumption on the consumer health and food perception during COVID-19. The study was conducted in 12 fast foods restaurants of 3 different universities in Jordan which are located in different places in Jordan. A desirable practice was given a score of one while no score will be allotted for an undesirable practice through using a standard questionnaire for all the food establishments. This was used to compare with the maximum score obtainable for that relevant operation and the percentage scores was calculated for each operation. Analysis of Variance (ANOVA) of the data was performed to study the significant differences at P ≤ 0.05 in all the evaluated properties among the food establishments in the different universities. The results showed that low percentage scores were obtained 68%, 75%, 32% and 56% for the production area, waste management, product evaluation (chemical, microbial) and food safety program application, respectively. Also, it was found that there were insignificant differences at P ≤ 0.05 among different food serving establishments in different universities in all the safety properties that were mentioned above. This means that all the food establishments in all the universities suffer from the same problems with the same degree. As a conclusion, more work should be done to produce safe food in the different food establishments that were studied in the different universities.

### P6 Synthesis of new potential beta adrenergic receptor agonist

#### Abdullah Saleh^1^, Zyad Abuelioah^2^

##### ^1^Al Ain University, UAE; ^2^The Hashemite University, Jordan

###### **Correspondence:** Abdullah Saleh (abdullah.saleh@aau.ac.ae)

*BMC Proceedings 2023*, **17(16):**P6

β-Amino alcohols (aryloxypropanolamine), are common β-Adrenergic blocking agents (β-blockers). are members of the large family of G-protein coupled receptors. β-Adrenergic blocking agents (β-blockers) are important in the treatment of many diseases in humans, such as hypertension, heart failure, cardiac arrhythmias, myocardial oxygen need, and to control dysrhythmia, thus, preventing long-lasting cardiac depression [1-3].

It is aimed to synthesize a library of β-Amino alcohols starting from epichlorohydrin and substituted 1,3-dicarbonyl compounds in 3 steps.

References

1. V. S. Borude, R.V. Shah and S. R. Shukla, Synthesis of β-amino alcohol derivatives from phenols in presence of phase transfer catalyst and lipase biocatalyst, Curr. Chem. Lett., 2, 1–12, 2013.
2. Paul W., Chi M., Richard J., William G., Ultra-Short-Acting fl-Adrenergic Receptor Blocking Agents. 1- (Ary1oxy) propanolamines Containing Esters in the Nitrogen Substituent, J. Med. Chem., 25, 1402-1407, 1982.
3. S. N. Louis , T. L. Nero, D . Iakovidis, F. M. Colagrande, G. P. Jackman,
4. W. J. Louis , β1- and β2-Adrenoceptor antagonist activity of a series of para-substituted N-isopropylphenoxypropanolamines, Eur. J. Med. Chem. 34 ,919−937 , 1999.

### P7 Application of physiological based pharmacokinetic model to study formulation effects of pharmacogentic drug

#### Muhammad Sarfraz, Rami Abu Jarad

##### Al Ain University, UAE

###### **Correspondence:** Muhammad Sarfraz (muhammad.sarfraz@aau.ac.ae)

*BMC Proceedings 2023*, **17(16):**P7

Background

The study used Quality by Design (QbD) approach that help to formulate the control release (CR) dosage form of drug undergoing pharmacogenetic variation inside human body. Dextromethorphan is used as a model drug that undergo polymorphic metabolism due to pharmacogentic variation as poor and extensive metabolism.

Methodology

The in silico Physiological based pharmacokinetic model (PBPK) was developed by incorporating the physiochemical, pharmacokinetic properties of model drug using GastroPlus® software. The PBPK model was run with standard dose of 30 mg Immediate release tablet and tested against its drug drug interaction (DDI) with quinidine at different concurrent doses. There simulated plasma profile results were compared with the existing clinical data to validate the PBPK model. This validated model was now simulated against different in vitro dissolution data to simulate its plasma profile in extensive and poor metabolism population. The formulation tested follow zero order release, Immediate release, sustained release and its combination designated as (F1 to F4) formulation.

Result

The simulated plasma profile showed the significance difference in extensive and poor metabolizer population in all the simulation. A gradual increase in the plasma DEM concentration was observed when quinidine was co-administered. Cmax, tmax and AUC0-24 varied depending on the formulations (F1–F4) within extensive and poor metabolism population.

Conclusion

The (QbD) approach along with in silico modeling help the formulation scientist to develop the optimal dosage formulation that can tailor the pharmacokinetic shape towards a desired drug plasma concentration.

### P8 Synthesis of 3’-deoxy-3’-acrylamide and cyanoacrylamide-containing ribonucleotide analogues as potential antiviral or anticancer agents

#### Ahmed Ibrahim, Abdullah Saleh, Tareq Abu-Izneid

##### College of Pharmacy, Al Ain University, Al Ain, UAE

###### **Correspondence:** Ahmed Ibrahim (ahmed.ibrahim@aau.ac.ae)

*BMC Proceedings 2023*, **17(16):**P8

Background

Nucleoside and nucleotide analogues (NAs) are a group of drugs that are mimics of the natural nucleosides and nucleotides [1–3]. They were of a remarkable importance in drug discovery, after zidovudine and abacavir emerged, proving a successful therapy against HIV in 1980s-1990s. Uses of NAs include antiviral, antiparasitic, and anti-cancer agents [1,2]. This study aims to synthesize novel adenosine, cytidine, and uridine analogues containing acrylamide and cyanoacrylamide at the 3’ carbon.

Method

Starting from the natural nucleosides mentioned above, chemical modification will be applied to modify the 3’-C, resulting in removal of 3’-OH and addition of acrylamide and cyanoacrylamide at the 3’-C. Once applied, a phosphate group is added to the 5’-OH, and masked to give prodrugs. The synthesized compounds are then going to be assessed for safety and biological activity against viruses and cancer.

Conclusion

The research is still in progress with no major results collected yet. However, it is believed that this study is going to provide new insight on whether or not the addition of acrylamide and cyanoacrylamide at the 3’-C of nucleotides will have a certain biological activity against certain diseases, which might be later considered as potential covalent therapeutic agents. This might help to further conduct other studies to investigate the addition of such groups for different nucleotides at different positions.

References

1. Li G, Yue T, Zhang P, Gu W, Gao LJ, Tan L. Drug Discovery of Nucleos(t)ide Antiviral Agents: Dedicated to Prof. Dr. Erik De Clercq on Occasion of His 80th Birthday. Molecules [Internet]. 2021 Feb 2 [cited 2022 Nov 6];26(4). Available from: https://pubmed.ncbi.nlm.nih.gov/33572409/
2. Meanwell M, Silverman SM, Lehmann J, Adluri B, Wang Y, Cohen R, et al. A short de novo synthesis of nucleoside analogs. Science (1979) [Internet]. 2020 Aug 7 [cited 2022 Nov 6];369(6504):725–30. Available from: https://www.science.org/doi/10.1126/science.abb3231
3. Seley-Radtke KL, Yates MK. The evolution of nucleoside analogue antivirals: A review for chemists and non-chemists. Part 1: Early structural modifications to the nucleoside scaffold. Antiviral Res [Internet]. 2018 Jun 1 [cited 2022 Nov 6];154:66. Available from: /pmc/articles/PMC6396324/

### P9 Community pharmacists perception towards an expanded role in practice

#### Amira SA Said^1,2^, Soha HS Aboueida^1^

##### ^1^Department of Clinical Pharmacy, College of Pharmacy, Al Ain University, Al Ain, Abu Dhabi, UAE; ^2^AAU Health and Biomedical Research Centre, Al Ain University, Abu Dhabi, United Arab Emirates

###### **Correspondence:** Amira SA Said (amira.ahmed@aau.ac.ae)

*BMC Proceedings 2023*, **17(16):**P9

Background

UAE Medical legislations have been changed to adapt to the modern substantial expansion of pharmacist role in practice. This worldwide shift in pharmacist's role has restructured their professional image and exerted more pressure for pharmacist involvement in direct patient care. This study aimed to address the perceived pharmacist's perception, current practice limitation and barriers that may affect their advanced services provision in the UAE.

Materials and Methods

A cross sectional survey using a self-administered 35 item questionnaire was conducted at Al Ain city, UAE. A convenient sample of 200 community pharmacists had completed the surveys over a period of 5 months. The objectives of the study were thoroughly explained to participants by the researchers and verbal consent was obtained. The data were analysed using SPSS descriptive statistics.

Results and Discussion

A total of 200 pharmacists participated in this study with mean±SD age of 36.5±5.9. Most participants agreed that the current pharmacists practice in the UAE is still to a large extent product oriented as pharmacists were mainly medication dispensers. This study has shown good pharmacists' perception towards expanded pharmacy practice concept, yet most pharmacists failed to engage properly in expanded practice services. This may be attributed to various obstacles faced in the community pharmacy settings. As shown form this study, the lack of self-trust, time, or enough pharmacy staff have been main limitations facing pharmacist in practice.

Conclusion

This study suggested that community pharmacists had sufficient awareness about the concept of extended pharmacy practice, yet they needed proper education, training and emphasis to properly implement better expanded patient care. However apparent lack of pharmacy facilities, or pharmacists' potential still remain possible obstacles to be tackled. Further work should focus on ways to overcome these barriers as pharmacists are particularly well-situated to be a much-expanded effective health contributors

Keywords

Pharmacist role –Chronic disease management - Medication use review – Perception - public health.

### P10 Thiamine, vitamin C and vitamin D mixture ameliorate hepatic injury in rat model of lipopolysaccharide-induced sepsis via down-regulation of NF-Κb

#### Yazan Ranneh^1^, Mahmoud Abu Ghoush^1^, Abdulmannan Fadel^2^

##### ^1^Department of Nutrition and Dietetics, College of Pharmacy, Al Ain University, United Arab Emirates; ^2^Sport and Exercises Sciences School, Faculty of Science, Liverpool John Moores University, Liverpool, UK

###### **Correspondence:** Yazan Ranneh (yazan.ranneh@aau.ac.ae)

*BMC Proceedings 2023*, **17(16):**P10

Background

Sepsis accompanied with elevated levels of endotoxin causes liver dysfunction with increased potential of mortality. This study was designed to elucidate the hepatic protective potential of thiamine (vitamin B1), ascorbic acid (vitamin C) and calciferol (vitamin D) mixture in Sprague Dawley rats challenged with lipopolysaccharide (LPS).

Methods

Eighteen Sprague Dawley male rats were randomly divided into three groups (control, LPS-treated with vitamin mixture, LPS-treated with saline). A mixture of thiamine (50 mg/Kg), vitamin C (500 mg/Kg) and vitamin D (200 ng/Kg) in 1 mL volume was administrated by oral gavage for 15 days consecutively. On day 15 and after 6 hours of intraperitoneal LPS injection (6 mg/Kg), blood and hepatic tissues were collected from all the animal groups.

Results

Vitamins mixture decreased the mortality rate of septic rats, aspartate aminotransferase, Alanine transaminase, alkaline phosphatase and total bilirubin levels in hepatic tissues. Using sandwich ELISA kits, vitamins mixture intake induced a significant reduction in serum TNF-α, IL-6, IL-1β and an increment in serum CAT, SOD and GSH. Western blot analysis demonstrated that vitamins mixture treatment constringed the expression of NF-κB p65, p38 MAPK and HMGB-1 in rats challenged with LPS. The histopathological observations due to LPS injection showed a significant improvement as a result of vitamins mixture treatment.

Conclusion

Thiamine, ascorbic acid and calciferol mixture ameliorated acute inflammation and could be potential adjuvant therapy for sepsis-induced hepatic injury.

### P11 Identification of potential hENT1 inhibitors using a combined approach of ligand-based and receptor-based virtual screening

#### Azza Ramadan^1,2^, Rose Ghemrawi^1,2^, Sedra Jamal^1^, Lama Abuamer^1^, Yusra Maher^1^, Mohammad Ghattas^1,2^

##### ^1^Department of Pharmaceutical Sciences, College of Pharmacy, Al Ain University, Abu Dhabi Campus, UAE; ^2^AAU Health and Biomedical Research Center, Al Ain University, Abu Dhabi, United Arab Emirates

###### **Correspondence:** Azza Ramadan (azza.ramadan@aau.ac.ae)

*BMC Proceedings 2023*, **17(16):**P11

Background

Human equilibrative nucleoside transporters (hENTs) are a family of integral proteins mostly found on cell plasma membranes. hENTs’ primary function is transporting vital nucleosides and nucleobases essential for DNA and RNA synthesis. Pharmacologically, the isoform hENT1 is an important therapeutic target, as inhibitors of hENT1 transport are cardio- and neuroprotective. However, current inhibitors are not clinically used due to their poor pharmacological profile. Hence, the overarching aim of this study is to utilize computer-aided drug discovery techniques to search for novel hENT1 inhibitors. Specifically, the objectives are a) to employ previously validated pharmacophores as filters prior to the intended virtual screening to identify potential hENT1 inhibitors, b) to develop an in vitro-based assay for assessment of hENT1 inhibition, and c) to evaluate the inhibition activity of the potential hENT1 inhibitors in vitro.

Methods

Pharmacophoric features were created based on standard hENT1 inhibitors. The validated pharmacophore was then used to screen a commercial drug-like ligand library. The identified candidate inhibitors were docked into the hENT1 pocket in a multi-step protocol using GLIDE and then ranked for a precise selection process. For the assessment of hENT1 inhibition, an MTT assay was utilized.

Results

Top-ranked compounds were visually inspected and limited to ten potential inhibitors. The criteria for selecting the compounds included the quality of interactions, the fitting into the target pocket, and their belonging to various scaffolds. Cytotoxicity assay using the H292 cell line was developed to evaluate the ten potential hENT1 inhibitors.

Conclusion

We successfully identified ten potential hENT1 inhibitors. An in vitro-based assay was established to assess the hENT1 inhibition activity of candidate inhibitors. These newly identified compounds can serve as novel therapeutic agents for cardiovascular and neurological disease treatment.

### P12 Antioxidant and antimicrobial properties of propolis from different geographic regions in UAE and its Applications in Food Safety

#### Hassan M Al Marzooqi^1^, Mohamed G Shehata^1,2^, Hanan S Afifi^1^, Saad H Masry^3,4^, Shabarinath Srikumar^5^

##### ^1^Food Research Section, R&D Division, Abu Dhabi Agriculture and Food Safety Authority (ADAFSA), Abu Dhabi P.O. Box 52150, United Arab Emirates

###### **Correspondence:** Hassan M Al Marzooqi (hassan.marzouqi@adafsa.gov.ae)

*BMC Proceedings 2023*, **17(16):**P12

Propolis is a resinous beehive product containing functional compounds and differs based on geographical region. Physicochemical properties of each propolis effectuate their uses within modern applications. Therefore, this study was aimed to evaluate the antioxidant and antimicrobial, of different propolis extracts (PEE) sourced from four geographical regions in UAE. Antioxidant potency was determined using DPPH. The phenolic (TPC) and flavonoid content (TFC) was calculated as gallic acid and catechol equivalents, respectively. We also identified Polyphenol compounds using RP-HPLC. Antimicrobial properties of propolis against pathogens were determined using agar well diffusion assay. Results of the antioxidant activity revealed that the highest activity was observed for wathba and kuwaitat propolis extract using DPPH assays with IC50 0.28±0.002 and 0.30±0.052 mg/ml, respectively. The antimicrobial activity against various pathogenic strains revealed that the propolis extracts of both wathba and kuwaitat exhibited the best antimicrobial activity against Klebsiella pneumoniae ATCC12296, Bacillus cereus ATCC 49064, Salmonella senftenberg ATCC 8400, Escherichia coli BA 12296, Yersinia enterocolitica ATCC 23715, Listeria monocytogenes ATCC 19116 and Campylobacter jejuni ATCC 700819. RP-HPLC analysis of ethanolic extracts of kuwaitat and wathba propolis revealed 23 polyphenolic compounds including phenolic acids and flavonoids. The predominant polyphenols were vanillic acid, caffeic acid, p-hydroxybenzoic acid, catechin, chlorogenic, syringic acid, p-coumaric acid, benzoic acid, rutin, o-cumaric acid, myricetin and kampherol. This study demonstrated the distinguishes between propolis sourced from different regions regarding their bioactive contents. Overall, kuwaitat and wathba propolis extract have antioxidant and antimicrobial effect with different spectrum and therefore, it might consider a potent candidate for treatment of several diseases. Also, propolis extract can be recommended as a preservative additive that can be applied to solve numerous food safety issues.

### P13 Development and in vitro evaluation of the controlled-release delivery system of xanthine filled in hard gelatin capsule size 00

#### Molham Sakkal, Mosab Arafat

##### College of Pharmacy, Al Ain University, Al Ain P.O. Box 64141, United Arab Emirates

###### **Correspondence:** Mosab Arafat (mosab.arafat@aau.ac.ae)

*BMC Proceedings 2023*, **17(16):**P13

Controlled drug delivery systems are well known to enhance patient compliance and reduce the dosing interval frequencies by sustaining the release of the drug molecules from the medication. The aim of this research work was to evaluate the release of xanthine derivatives molecules over a long period of time by developing a new matrices formulation system using different ratios of triblock copolymer materials incorporated with different percentages of other amphiphilic additives. various controlled release matrices formulation systems were developed and prepared using a number of series with different ratios of the copolymer to the mixture. Besides, the fusion method was used for matrices system preparation. The drug release of xanthine was evaluated over 12 hrs using a number of incubated media with different pH values. UV spectrophotometer was used in order to measure the amount of drug release with UV-detecting light adjusted at 272 nm. Results showed a significant gradual decrease in drug release rate upon increasing the portion of copolymers mixture in the matrix system, respectively. The delay in the drug release over a long period of time might be attributed to the amphiphilic natures of the copolymeric mixture in the matrices system. The hydrophobic part of copolymeric materials in the matrix system was slowly dissolved whereas the hydrophilic part was presumed to swell and form a gel layer upon exposure to the dissolution media which promotes the diffusion of the drug molecule throughout the matrix system. Therefore, variations of copolymer mixture incorporated in the system can vary the drug release rate over a controlled period of time. In conclusion, the incorporation of a high portion of the triblock copolymer in the matrix’s mixture was a successful approach to control the release of BCS Class I drug. Moreover, this matrix system is easy to prepare and is considered a promising system for drug delivery.

### P14 Evaluating the use of evidence-based medicine in health care in the United Arab Emirates

#### Mohammad M Al Ahmad

##### Al Ain University, UAE

###### **Correspondence:** Mohammad M Al Ahmad (mohammad.alahmad@aau.ac.ae)

*BMC Proceedings 2023*, **17(16):**P14

Background

Evidence-based medicine (EBM) is an evolving field of medicine and medical diagnostics. The challenges in this field make it necessary for healthcare providers (HCPs) to be familiar with the current state of knowledge in the medical literature in order to make the most appropriate decisions in the diagnosis and treatment of diseases. The aim of this study is to investigate the attitudes of healthcare providers in medical institutions in the United Arab Emirates (UAE) towards EBM.

Methods

A structured questionnaire with cross-sectional observation was designed and distributed to healthcare professionals in the UAE. All participants were volunteers. The questionnaire included 5 domains and 23 different Likert scale questions to assess the knowledge, attitude, advantages, disadvantages, and limitations of using EBM in practice. The reliability and validity of this study were assessed using Cronbach's alpha. The SPSS system was used for descriptive and statistical analysis.

Results

There were 398 health care providers who participated in this study; 232 (58.3%) were male and 166 (41.7%) were female. The majority of participants had more than 5 years of experience. 253 (63.5%) participants were physicians and 145 (36.5%) participants were pharmacists. More than half of physicians and pharmacists had a positive attitude toward EBM knowledge and practice. 40% of physicians and pharmacists rated the benefits of implementing EBM in practice as excellent. Challenges such as lack of time and cost were also cited by participants.

Conclusion

Health professionals' positive attitudes toward EBM need to be supported to improve its adoption and dissemination. The challenges and limitations identified in the results could be used to develop new techniques to resolve them or at least reduce their impact in practice, thereby promoting acceptance of EBM in practice among health professionals.

### P15 In silico evaluation and design of allosteric SARS-CoV-2 Main Protease (Mpro) inhibitors via structure-based drug design

#### Lara Alzyoud^1,2^, Radwa E. Mahgoub^1,2^, Fedaa Mohamed^3,4^, Bassam R. Ali^3,4^, Wael Rabeh^5^, Juliana Ferreira^5^, Noor Atatreh^1,2^, Mohammad A. Ghattas^1,2^

##### ^1^College of Pharmacy, Al Ain University, Abu Dhabi 64141, United Arab Emirates; ^2^AAU Health and Biomedical Research Center, Al Ain University, Abu Dhabi 64141, United Arab Emirates ^3^Department of Genetics and Genomics, College of Medicine and Health Sciences, United Arab Emirates University, Al-Ain 15551, United Arab Emirates; ^4^Zayed Centre for Health Sciences, United Arab Emirates University, Al-Ain 15551, United Arab Emirates ^5^Science Division, New York University Abu Dhabi, Abu Dhabi 129188, United Arab Emirates

###### **Correspondence:** Mohammad A. Ghattas (mohammad.ghattas@aau.ac.ae)

*BMC Proceedings 2023*, **17(16):**P15

With more than 635 million confirmed cases and near to 6.6 million deaths (as per WHO records), the COVID-19 pandemic is far from over and continues to impose challenges on almost every aspect of our daily lives. The Mpro enzyme is essential for disease progression and plays an integral role in the SARS-CoV-2 virus's life cycle [1]. So far, substantial research has been conducted to develop novel Mpro inhibitors, the majority of which target the enzyme's catalytic pocket. However, only a little research has been done on the Mpro allosteric site, which we want to target in our work. Allosteric sites are believed to be less prone to mutations and more conserved among different coronavirus strains; therefore, they make an attractive target for potential wide-spectrum anti-corona viral agents. In particular, sites on the dimerization interface appear to be important, as blocking dimerization is believed to abolish the activity of the protease. Hence, we evaluated all putative allosteric sites on the Mpro dimer structure [2]. Two cavities (i.e. sites #2 and #5) turned to possess a druggable character and were therefore predicted to bind drug-like molecules. These sites, which are located at and near the dimer interface, were targeted through computer-aided drug design by structure-based virtual screening of ~5 million ligands. After extensive filtration, docking, and post-docking analyses, 44 compounds were selected for experimental testing. CL02, our top hit, is a non-competitive inhibitor of Mpro with a Ki of 115 μM and to our knowledge, the highest ligand efficiency for an allosteric Mpro inhibitor. Moreover, CL02 has a small size (~355 Da) and drug-like characteristics making it a promising lead candidate for future developments. The findings from this work may aid in the ongoing fight against COVID-19 and, potentially, against future coronaviruses-related diseases.

References

1. Jin Z, Du X, Xu Y, Deng Y, Liu M, Zhao Y, et al. Structure of Mpro from SARS-CoV-2 and discovery of its inhibitors. Nature [Internet]. 2020 Apr 9 [cited 2021 Sep 26];582(7811):289–93. Available from: https://www.nature.com/articles/s41586-020-2223-y
2. Alzyoud L, Ghattas MA, Atatreh N. Allosteric Binding Sites of the SARS-CoV-2 Main Protease: Potential Targets for Broad-Spectrum Anti-Coronavirus Agents. Drug Des Devel Ther [Internet]. 2022 [cited 2022 Nov 28];16:2463–78. Available from: https://pubmed.ncbi.nlm.nih.gov/35941927/

### P16 The impact of health belief model on nonadherence with medication therapy in the treatment of hypertensive and the associated factors in an outpatient clinic.

#### Faris El-Dahiyat^1,2^, Ammar Abdulrahman Jairoun^3,4^, Sabaa Saleh Al-Hemyari^4,5^, Abdullah Elrefae^6^, Mohammed Alsbou^7,8^

##### ^1^Clinical Pharmacy Program, College of Pharmacy, Al Ain, Al Ain, United Arab Emirates; ^2^AAU Health and Biomedical Research Canter, Al Ain University, Abu Dhabi, United Arab Emirates; ^3^Health and Safety Department, Dubai Municipality, Dubai, UAE; ^4^School of Pharmaceutical Sciences, Universiti Sains Malaysia (USM), Pulau Pinang 11500, Malaysia; ^5^Pharmacy Department, Emirates Health Services, Dubai, United Arab Emirates; ^6^Colchester General Hospital, Essex, Colchester CO4 5JL, UK; ^7^Department of Pharmacology, Faculty of Medicine, Mutah University, Jordan; ^8^Faculty of Medicine, Ajman University, Ajman, UAE

###### **Correspondence:** Faris El-Dahiyat (faris.dahiyat@aau.ac.ae)

*BMC Proceedings 2023*, **17(16):**P16

Background

Around the world, hypertension is a major factor in cardiovascular disease and premature death. Non-adherence to the prescribed antihypertensive medication lead to uncontrolled high blood pressure which results in serious complications.

Objectives

To determine the nonadherence to antihypertensive treatment and the associated factors with the aid of the health belief model (HBM) [1-3].

Methods:

A cross-sectional analytical study was carried on at an outpatients’ clinic in Jordan enrolled 660 patients with hypertensive. The questionnaire included information on demographics, lifestyle factors and five-point Likert type HBM questionnaire.

Results

The level of nonadherence to antihypertensive treatment in the current study was 60.5%. The average age (±SD) of the patients was 45.5 (±4.2) years, while the median value for hypertension duration was two years. Five HBM constructs explained variance for 35.8% in nonadherence to antihypertensive treatment and the prediction accuracy was 79.4%, after adjustment for gender, age and duration of condition. In the current study, significantly reduced risks of nonadherence were observed in cue to actions [OR=0.49(0.28-0.70), p=0.005], higher levels of perceived benefits from medicine use [OR=0.45(0.26-0.62),p=0.001]. In contrast, increased risk of nonadherence were observed in perceived barrier [OR=3.14(2.26-4.32), p<0.001], perceived severity [OR=6.20(3.91- 7.13),p<0.0001] and perceived susceptibility [OR=5.02(2.60-8.65), p<0.001]. Moreover, reduced levels of nonadherence were significantly higher among higher education holders [OR=0.21(0.13-0.13), p<0.001] and among participants who had higher income [OR=0.72(0.48 0.91), p<0.001].

Conclusion: Based on the study, it can be concluded that non-adherence with antihypertensive therapy is significantly high, while HBM is reliable for assessing non-adherence with therapy in hypersensitive patients. Higher levels of severity, sensitivity, and perceived barriers are significant predictors of non-adherence with antihypertensive therapy. Intervention strategies may be influenced in clinical practice by the correlation between risk factors, HBM components, and non-adherence to antihypertensive medication.

References

1. Joho AA. Using the Health Belief Model to Explain the Patient's Compliance to Anti-hypertensive Treatment in Three District Hospitals - Dar Es Salaam, Tanzania: A Cross Section Study. East Afr Health Res J. 2021;5(1):50-58. doi: 10.24248/eahrj.v5i1.651. Epub 2021 Jun 11. PMID: 34308245; PMCID: PMC8291213.
2. Sulat, J. S., et al. "The validity of health belief model variables in predicting behavioral change: a scoping review." Health Education 118.6 (2018): 499-512.
3. Al-Noumani H, Wu JR, Barksdale D, Sherwood G, Alkhasawneh E, Knafl G. Health beliefs and medication adherence in patients with hypertension: a systematic review of quantitative studies. Patient education and counseling. 2019 Jun 1;102(6):1045-56.

### P17 Targeting the post-translational modification “methylation” for treating breast cancer

#### Aya Al Qassem^1,2^, Azza Ramadan^1,2^, Rose Ghemrawi^1,2^

##### ^1^ College of Pharmacy, Al Ain University, Abu Dhabi 64141, United Arab Emirates; ^2^ AAU Health and Biomedical Research Center, Al Ain University, Abu Dhabi 64141, United Arab Emirates

###### **Correspondence:** Rose Ghemrawi (rose.ghemrawi@aau.ac.ae)

*BMC Proceedings 2023*, **17(16):**P17

Breast cancer is one of the most prevalent cancers and the leading cause of cancer-related death among women. Its annual incidence and related death rate are steadily increasing worldwide. Protein methylation, a post-translational modification, regulates gene transcription, RNA processing, translation, signal transduction, DNA damage response, and the cell cycle. It is also known that aberrant methylation contributes to the malignant transformation of cells by silencing critical tumor suppressor genes. It was found that methyltransferase inhibitors reactivate silenced tumor suppressor genes and result in tumor growth arrest. Therefore, clinical studies suggested the inhibition of methyltransferases as a promising target in cancer therapy. In order to find a promising candidate methyltransferase inhibitor as an anti-breast cancer therapeutic agent, our project aims to study first the effect of methylation inhibition on breast cancer cell lines’ proliferation, viability, apoptosis and migration using the global methyltransferase inhibitor, Adenosine dialdehyde (AdOx). Therefore, the wound healing assay, MTT and apoptosis tests were performed in presence and absence of AdOx on MCF-7 and MDA-MB 231 cell lines. Interestingly, breast cancer cells’ viability, proliferation and migration were dramatically reduced, apoptosis was increased. These results confirm that inhibiting the methylation is a potential treatment of breast cancer and that finding new pharmacological methyltransferase inhibitors is crucial.

### P18 Synthesis and in-vitro characterization of thiolated ultra-low molecular weight polymeric conjugates for mucosal drug delivery

#### Arshad Mahmood

##### College of Pharmacy, Al Ain University, Abu Dhabi Campus, Abu Dhabi, United Arab Emirates

###### **Correspondence:** Arshad Mahmood (arshad.mahmood@aau.ac.ae)

*BMC Proceedings 2023*, **17(16):**P18

Background

The aim of current study was to extend the application of thiolation to the ultra-low molecular weight polymers. These mucoadhesive conjugates might provide the opportunity to be used in high concentrations without affecting much to the viscosity when intended for freely flowing liquid preparation.

Results

A copolymer, poly(acrylic acid-co-maleic acid) [PAAMA], MW~3kDa, was thiolated by two different thiol bearing moieties, cysteamine and cysteine separately, using well-known carbodiimide/N-Hydroxysuccinimide based amide formation scheme. The covalent bonding was qualitatively confirmed by fourier transform infrared spectroscopy and quantitatively via Ellman’s reagent, that revealed PAAMA conjugates exhibited good thiolation, being 3134.30 μmol/g for PAAMA-cysteamine and 1268.37 μmol/g of thiol groups for PAAMA-cysteine. The synthesized conjugates were found non-toxic (cell viability > 85%) over Caco-2 cells over a period 3 h of exposure. The water carrying capacity was observed in the presence of carbopol, as diluent to formulate a disc and the outcomes demonstrated 1.48- and 1.35- folds higher swelling for PAAMA-cysteamine and PAAMA-cysteine compared to PAAMA, respectively. Mucoadhesive potential measured on the freshly excised rabbit mucosa via rotating cylinder method demonstrated 4.1- and 2.4-folds higher residence time for PAAMA-cysteamine and PAAMA-cystein compared to PAAMA, respectively. Moreover, rheological measurements of aqueous polymer solution and as polymer/mucus mixtures illustrated only mild increase in dynamic viscosity (ƒ*) at a concentration of 5%, compared to blank aqueous and unmodified polymer solutions.

Conclusion

Based on our results it can be concluded that the low molecular weight thiolated conjugates appear promising mucoadhesive materials for freely flowing dosage forms and are able to extend the residence time over the target mucosa with minimum increase in viscosity.

### P19 Comparison between branded and generic Glimepiride 1 mg tablets using in vitro evaluation and thermal analysis assessment methods

#### Mosab Arafat^1^, Anna Esmaeil^1^, Salah AbuRuz^2^

##### ^1^College of Pharmacy, Al Ain University, Al Ain P.O. Box 64141, United Arab Emirates; ^2^Department of Pharmacology and Therapeutics, College of Medicine and Health Sciences, United Arab Emirates University, Al Ain, Abu Dhabi, UAE

###### **Correspondence:** Mosab Arafat (mosab.arafat@aau.ac.ae)

*BMC Proceedings 2023*, **17(16):**P19

Generic medications are considered a suitable alternative for brand medications since they are bioequivalent to each other. Yet, the quality and purity of generic medications are still debatable. The aim of this study was to evaluate and compare the quality and performance of a generic Glimepiride tablet 1 mg and a brand one using chemical and thermal analytical instruments. Quality control assessments, and in vitro dissolution tests were carried out to assess the physiochemical properties and the drug release rate for both products. Additionally, several analytical techniques were used, namely: Thermogravimetric analysis (TGA), differential scanning calorimetry (DSC), scanning electron microscopy (SEM), Fourier-Transform infrared (FT-IR), confocal microscope with Raman spectroscopies and X-ray Diffraction (X-RD). Results showed that there were some variations between brand and generic medications. In terms of tablet hardness and disintegration, generic medications were almost different from the brand one in mean resistance force, on the contrary, the brand product took almost more time to disintegrate completely. The release rate of the generic drug was faster compared to the brand when was incubated in three different pH media. In terms of thermal analysis, DSC revealed a variation in endothermic peak, whereas a sharp endothermic peak for generic and brand medications was slightly different, additionally, TGA showed almost less in weight loss for the generic compared to brand. Other tools and assessments presented almost identical outcomes. The obtained variations between generic and brand could be attributed to the existence of different excipients. Another possibility of variation could be due to the interaction between excipients, which was represented by the use of a spectroscope. In conclusion, the variations between the generic and brand was existed and the utilization of chemical and thermal analysis was useful in determining these variations.

### P20 UAE pharmacists’ attitudes toward including diagnosis or clinical indication on prescription orders

#### Amar M Hamrouni

##### Al Ain University

###### **Correspondence:** Amar M Hamrouni (amar.hamrouni@aau.ac.ae)

*BMC Proceedings 2023*, **17(16):**P20

Background

Currently, pharmaceutical laws in UAE do not require that a diagnosis or drug indication to be included in prescriptions. Medication safety advocates, regulatory agencies, and professional pharmacy organizations have advocated for the inclusion of such information on prescriptions to improve safety and clinical effectiveness.

In the USA as another example currently state and federal laws do not require the indication for the use of drugs on prescription orders. A prescription in Arizona for instance should contain the date of issue, name and address of the patient, refills authorized, if any, name, address and telephone number of the prescribing medical professional, the name, strength, dosage form and quantity of the drug ordered and directions for use [1]. Pharmacists by law must prospectively evaluate the prescribed medication to ensure its appropriateness, and that it is medically necessary, and not likely to produce in adverse reactions [2]. Without the clinical diagnosis pharmacist may make an educated guess, or may spend long time calling the prescribers for some clarifications regarding the medication prescribed [3].

Aim

To gauge the thoughts about requiring either diagnosis or clinical indication on prescription orders in UAE and to determine whether UAE pharmacists support such practice, which aims to improve the counselling experience and allow the pharmacists to do successful counselling.

Methods

This was a mixed-method study with data obtained from questionnaires collected from community and hospital pharmacies, conducted in Al Ain, UAE. The questionnaire consisted of 20 questions extracted from the participant's experiences and opinions about the topic. A total of 150 licensed pharmacists were included in the study.

Main Results

The majority of pharmacists (92; 61.3%) included in the study supported having the diagnostic and clinical indication to be included in the prescription. Interestingly, (34, 22.7%) and 24 (22.7%) reported having either the diagnosis or clinical indication respectively.

Conclusion

Inclusion of diagnostic or clinical indication in a prescription would help to better educate and empower individuals about their medications, improve communication within the healthcare team, and increase the speed and efficiency of prescribing by presenting drug choices for that indication.

Keywords

Medication Errors, Pharmacists, Patient Safety, United Arab Emirates, Diagnosis, Clinical indication, Community pharmacy, hospital pharmacy.

References

1. Arizona Revised Statutes 32-1968, subsection C. Accessed November 22, 2022.
2. Omnibus Budget Reconciliation Act of 1990 (OBRA-90; Pub.L. 101-508, 104 Stat. 1388, enacted November 5, 1990).
3. Odukoya OK, Stone JA, Chui, MA. How do community pharmacies recover from e-prescription errors?. Res Social Adm Pharm. 2014:837-852.

### P21 The prevalence of stimulant and nutritional supplement usage among students at Abu Dhabi Universities in gyms

#### Majed El-Saleh^1^, Ziyad Ellala^1^, Balkees Abuawad^2,3^, Shaden Al Atassi^2^

##### ^1^College of Education, Humanities and Social Sciences, Al Ain University, Abu Dhabi, United Arab Emirates; ^2^College of Pharmacy, Al Ain University, Abu Dhabi, United Arab Emirates; ^3^AAU, Health and Biomedical Research Center, Al Ain University, Abu Dhabi, United Arab Emirates

###### **Correspondence:** Majed El-Saleh (Majed.Elsaleh@aau.ac.ae)

*BMC Proceedings 2023*, **17(16):**P21

Background:

Students from universities in Abu Dhabi used stimulants and nutritional supplements at gyms. The study sought to pinpoint variations in prevalence percentages based on the variables (number of years of practice, level of education, age and gender) and other variables.

Materials and methods

512 male and female students from various colleges made up the study's sample, which was selected at random. The questionnaire served as a data collection tool for the descriptive survey method, which was utilized to obtain the results.

Results

Findings revealed that (46 %) of students who use nutritional supplements take them as tablets on a rate of (30.9%), compared to (9.8%) of those who use stimulants and take them as injections on a rate of (6.3%). According to the statistics, 8.2% of the study sample's supplement and stimulant users report using them for longer than a year. The findings also showed that (91.2%) of students can tell the difference between nutritional supplements and stimulants. The first year category attained the greatest rate, for the age variable. However, the gender variable was (52.1%) for males and (9634.7) for females. According to the years of physical activity variable, the use of nutritional supplements and stimulants was the most.

Conclusions

The recommendations encourage providing supervision for gyms and nutrition centers and require doctor's approval in prescribing nutritional supplements and stimulants also conducting educational courses and workshops for trainers and gyms supervisors to limit the spread of the sale of stimulants and supplements.

Keywords

Nutritional supplements, stimulants, gyms, sports nutrition, health awareness, health culture.

### P22 Phones microbial contamination in UAE during COVID-19 pandemic

#### Kawthar Kayed^1,2^, Rose Ghemrawi^1,2^

##### ^1^College of Pharmacy, Al Ain University, Abu Dhabi Campus, United Arab Emirates; ^2^AAU Health and Biomedical Research Center, Al Ain University, Abu Dhabi, United Arab Emirates

###### **Correspondence:** Rose Ghemrawi (rose.ghemrawi@aau.ac.ae)

*BMC Proceedings 2023*, **17(16):**P22

Background

91.69% of the population own a phone without considering the fact that on these devices, microbes can accumulate and transmit microorganisms. Our study investigated the bacterial contamination of mobile phones in Abu Dhabi, UAE.

Methods

This cross-sectional study involved 100 participants. A questionnaire was used to gather sociodemographic and phone usage data, followed by swabbing mobile phones for microbiological testing.

Results

Swabbing was taken during the COVID-19 pandemic; therefore, 41% of participants cleaned their mobile phones daily by using wipes or alcohol. However, 100% of participants had a mobile phone contaminated by bacteria such as S. aureus, CoNS, micrococcus spp, E. coli, bacillus spp, streptococcus spp, Citrobacter spp, proteus spp, enterococcus spp, klebsiella, pseudomonas and Actinobacteria. Most of these potentially pathogenic bacteria were resistant to ampicillin, Ceftazidime, and cefotaxime.

Conclusion

The frequency of disinfecting and cleaning hands and phones by our participants was not enough. Individuals need to make sure to have excellent hand hygiene and disinfect their mobile phones frequently.

### P23 Effect of diminazene, an angiotensin converting enzyme 2 activator, on fructose-induced hypertension in rats

#### Yousuf M. Al Suleimani, Aly M Abdelrahman, Priyadarsini Manoj, Mohammed Ashique, Badreldin H Ali

##### Sutan Qaboos University, College of Medicine and Health Sciences, Department of Pharmacological and Clinical Pharmacy, Oman

###### **Correspondence:** Yousuf M. Al Suleimani (yousufm@squ.edu.om)

*BMC Proceedings 2023*, **17(16):**P23

The aim of the present study was to investigate the effect of diminazene, an angiotensin converting enzyme 2 (ACE-2) activator on fructose-induced hypertension. Rats were fed 60% fructose diet for 8 weeks in the absence or presence of diminazene (15 mg/kg/day) in weeks 6-8. Blood pressure was measured by the tail cuff methods. Blood samples were collected to measure fasting plasma insulin, triglycerides (TG), cholesterol (TC), LDL-C, HDL-C, tumor necrosis factor-alpha (TNF-α), interleukin-6 (IL-6), malondialdhyde (MDA) levels, catalase and superoxide dismutase (SOD) activities. Fructose increased blood pressure, fasting plasma insulin, TG, TC, LDL-C, interleukin 6, MDA, and decreased catalase and superoxide dismutase (SOD) activities. Diminazene significantly reduced fructose-induced hypertension. Diminazene did not significantly affect fructose induced hyperinsulinemia or hyperlipidemia. Diminazene did not affect inflammatory markers but attenuated fructose induced oxidative stress. In conclusion, the present study showed that diminazene was partially successful in reversing fructose-induced hypertension but not hyperinsulinemia or hyperlipidemia.

### P24 Synthesis of highly potent Schiff based derivatives from primary amine drugs and aldehydes: New vistas in medicinal chemistry

#### Saad Touqeer^1^, Umair Ikram Dar^2^, Kishwar Sultana^2^

##### ^1^Al Ain University AD campus, UAE; ^2^Department of Pharmacy, The University of Lahore, Lahore, Pakistan

###### **Correspondence:** Saad Touqeer (saad.touqeer@aau.ac.ae)

*BMC Proceedings 2023*, **17(16):**P24

Schiff bases are generally synthesized by reacting primary amines with various aldehydes. Such derivatives show more potent activities as compared to the parent drugs [1]. Furthermore, a variety of metal complexes can be conveniently obtained in a single step. These metal complexes further extend the therapeutic scope of such drugs [2]. Herein, we discuss the one-pot synthesis and biological evaluation of various Schiff bases obtained from a plethora of aldehydes and amine or amide based drugs. The structures were confirmed using NMR and IR spectroscopies. The imines were complexed with Cu, Zn, Fe, Mg and Mn ions using their inorganic salts. Atomic absorption and X-Ray crystallographic analysis was carried out to confirm the structure of the metal complexes. Docking studies were carried out using AutoDock 4.2 showing scores significantly higher than that of standard drug compounds. Biological assays such as antibacterial, antifungal, antioxidant and anticancer were carried out with most of the derivatives exhibiting significantly higher activities than those of the original drugs. Results suggest the synthetic analogues to be of superior therapeutic value and potential for advanced preclinical studies and optimizations.

### P25 Machine learning algorithms and computational validation of CYP2C9 polymorphisms in predicting therapeutic outcomes of warfarin.

#### Kannan Sridharan^1^, Suchetha Manikandan^2^, George Priya Doss^3^, Rashed Al Banna^4^

##### ^1^Department of Pharmacology & Therapeutics, CMMS, Arabian Gulf University, Kingdom of Bahrain; ^2^Centre for Healthcare Advancement, Innovation and Research, Vellore Institute of Technology, Chennai, India; ^3^School of Bio Sciences and Technology, Vellore Institute of Technology, Vellore, India; ^4^Department of Cardiology, Salmaniya Medical Complex, Manama, Kingdom of Bahrain

###### **Correspondence:** Kannan Sridharan (skannandr@gmail.com)

*BMC Proceedings 2023*, **17(16):**P25

Background

Warfarin, a commonly used anti-coagulant drug is influenced by Cytochrome P450 (CYP) enzymes, particularly CYP2C9. Machine learning algorithms (MLAs) have been identified to have a great potential in personalized therapy. We carried out the present study to evaluate MLAs in predicting the key outcomes of warfarin therapy and validated the key predictor variable using bioinformatics tool.

Methods

A cross-sectional study was carried out in adults of either gender, non-smoking status receiving warfarin. Allele discrimination method for estimating the single nucleotide polymorphisms (SNPs) in CYP2C9 (rs1799853 and rs1057910), VKORC1 (rs9923231), and CYP4F2 (rs2108622). Genetic and clinical variables were used for predicting poor anticoagulation status (ACS) and warfarin stable dose. Logistic regression, support vector machine (SVM), random forest (RF), decision tree algorithms (DCA) were used for evaluating the ACS and linear regression, RF, DCA, SVM, and artificial neural network were evaluated for warfarin stable dose. Area-under-the-curve (AUC) was used as the measure of predictive accuracy for ACS and root mean square error (RMSE) for stable dose. We used advanced computational methods for examining the structural and functional consequences of SNPs in the CYP2C9 gene.

Results

We included data from 205 participants and observed no significant difference between the training and testing cohorts. Support vector machine with linear Kernel (AUC = 0.67) and linear regression (RMSE = 14.2 mg/week) was observed with the best predictive accuracy for poor anticoagulation status and stable warfarin dose, respectively. CYP2C9was observed to be the most important predictor for both outcomes. Computational validation methods confirmed the altered structural activity, stability, and impaired functions of protein products of CYP2C9 SNPs.

Conclusion

We have evaluated various MLAs in predicting the key outcome measures associated with warfarin and observed CYP2C9 to be the most important predictor variable. Computational validation model has also corroborated the reduced functional abilities of key SNPs in CYP2C9. A prospective study validating the MLAs is urgently needed.

### P26 Machine learning in predicting the risk factors and mortality in COVID-19 patients

#### Noor Salmeh^1^, Sedra Jamal^1^, Alin Alkawarit1, Asim Elnour^1^, Taima Alqudah^1^, Abdullah Shehab^2^

##### ^1^College of Pharmacy, Al Ain University, Abu Dhabi, UAE; ^2^Emirates Medical Association (EMA), UAE

###### **Correspondence:** Asim Elnour (asim.ahmed@aau.ac.ae)

*BMC Proceedings 2023*, **17(16):**P26

Background

The high public health concern for all nations is the coronavirus disease 2019 (COVID-19) pandemic brought on by the SARS-Cov2 virus has a high case fatality, and is linked to several clinical manifestations. For COVID-19 patients who are severely sick, predicting death and figuring out outcome determinants are vital. To create prediction models and simplify clinical phenotypes, multivariate and machine learning techniques might be applied.

Methods

A literature review of studies conducted on machine learning in predicting the risk factors and mortality in COVID-19 patients.

Results

Between the survivors and non-survivors, there were notable differences in the baseline characteristics. The area under the receiver operating characteristic curve (AUROC), that was used to compare the models’ performance, in all the reviewed 8 papers was > 0.85 suggesting that these models ware accurate enough to discriminate the deceased outcome of patients.

Conclusion

The studies reviewed showed the use of machine-learning-based approaches to predict hospital mortality in COVID-19 patients, the identification of the most significant predictors as well as the distinction between COVID-19 survivors at high- and low-risk.

### P27 Zinc attenuates the acute renal damaging effects of Hydroxychloroquine in adult male Albino rats

#### Nihal A Ibrahim^1^, Manal A Buabeid^2^, Kadreya E Elmorshedy^3^

##### ^1^College of Pharmacy and Health Sciences, Ajman University, UAE; ^2^Fatema College of Health Sciences, Abu Dhabi, UAE; ^3^College of Medicine, Tanta University, Egypt

###### **Correspondence:** Nihal A Ibrahim (n.ibrahim@ajman.ac.ae)

*BMC Proceedings 2023*, **17(16):**P27

It is reported that long term exposure to Hydroxychloroquine (HCQ) might increase the susceptibility of acute kidney injury by disrupting the autophagy-lysosomal pathway. The present work was conducted to study the hypothesis of a protective role of Zinc. A total of 40 normal adult male albino rats were used and divided randomly into 4 groups. Group 1 (Control group), Group II (HCQ treated group), Group III (Zinc treated group), Group IV (HCQ and Zinc treated group). After completion of the experiment period, all rats were sacrificed and renal tissue samples were processed twenty-four hours at the end of the experiment for both histological and immune-histochemical studies. Renal stained sections revealed that HCQ induced glomerular degeneration with reduced Bowmen’s capsular space, apoptosis and hydropic degeneration of renal tubules with excessive fibrosis in the wall of blood vessels and capsules. However, combination of HCQ with Zinc ameliorated these damaging effects as it displayed normal glomerular and tubular architecture. Quantitative analysis showed highly significant increase in the areas of fibrosis in group II compared to other groups (p<0.05.) These findings represent a valuable tool for Zinc-based therapy in the future after clinical trials to adjust the dose and ensure patient’s safety.

### P28 Evaluation of pasta enriched with Spirulina platensis microalgae: (2) Medicinal and biological evaluation

#### Gamali A El-Sharnouby^1^, Mahmood Abughoush^2^

##### ^1^Food Science and Technology Department, Collage of Agriculture, Al-Azhar University, P.O. Box 11884, Nasr City, Cairo, Egypt; ^2^Science of Nutrition and Dietetics program, College of Pharmacy, Al Ain University, P.O. Box 64141, Abu Dhabi, UAE.

###### **Correspondence:** Gamali A El-Sharnouby (Gamali59@azhar.edu.eg)

*BMC Proceedings 2023*, **17(16):**P28

Spirulina platensis microalgae is considered as a valuable source of antioxidants and Phyto nutritive compounds. Spirulina platensis is the most commonly available and widely used genus, which has been widely studied in different areas, including the food industry and medicine. One of its species, Spirulina platensis or its extract showed therapeutic properties, such as the ability to prevent cancers, decrease blood cholesterol level, reduce nephrotoxicity of pharmaceuticals and toxic metals, and provide protection against the harmful effect of radiation. Therefore, the aim of the present study is to determine antioxidant activity of Spirulina platensis powder and its pasta products against Liver disease in experimental Rats. The pasta was supplemented by adding Spirulina platensis powder at different levels (5, 10, 15 and 20 %). The results indicated a prospective result on reducing the liver damage which be indicated through ALT, AST, ALP, Albumin, creatinine, and total bilirubin in CCL4 intoxicated rats’ blood after the end of experiments. However, liver glutathione (GSH) concentration was markedly decreased as compared to control group. Additional studies are necessary to test the application of Spirulina Platensis in other contexts.

Keywords

Spirulina enrichment; green pasta; Liver enzymes; experimental Rats

### P29 Pharmaceuticals regulatory and administrative control bodies in UAE legislations

#### Ahmad A. Al Dalaien^1^, Mohammad Alkrisheh^2^, Faris El-Dahiyat^3^

##### ^1^College of Law Mutah University, Al Karak, Jordan; ^2^College of Law, Al Ain University, Al Ain, UAE; ^3^College of Pharmacy, Al Ain University, Al Ain P.O. Box 64141, UAE

###### **Correspondence:** Mohammad Alkrisheh (mohammad.alkrisheh@aau.ac.ae)

*BMC Proceedings 2023*, **17(16):**P29

Background

Regulatory affairs in pharma ensure all regulations and laws are followed. These include intellectual property rights to protect drug manufacturers' research, safety standards to protect the public from harmful side effects, restrictions on marketing drugs to the public, and rules regarding how drugs may be prescribed and distributed. This study aims to assess the administrative control bodies in preserving pharmaceutical preparations before they are circulated in UAE law through the legal means these administrative bodies resort to. The research problem is the multiplicity of bodies that undertake the control task and the insufficiency of legislation governing pharmaceutical safety control in the UAE legislation.

Method

This study uses an analytical approach by analyzing the legal texts governing the preventive measures to maintain the drug's safety, how to implement them, and the competent authorities to impose them so that we can determine the effectiveness of the measures taken by examining the approved mechanism for monitoring these authorities.

Results

There is an urgent need to unify the regulatory authorities with one entity entrusted with controlling the drug before it is put into circulation to ensure its safety. Its dispersion contributed to encouraging pharmaceutical institutions to practices that violate the law. Moreover, finding comprehensive legislation for everything related to pharmaceuticals protection and control. In addition to increasing deterrent penalties for anyone who violates public health standards in the production of medicine.

Conclusion

The UAE Pharmaceutical regulation needs to be revised to unify the regulatory authorities, and the legislation needs to be updated to cover all aspects of pharmaceutical products manufacturing and distribution.

### P30 Synthesis of mixed α,α-dihaloketones with potent anticancer activity using magnesium carbenoids

#### Vittorio Pace^1^, Saad Touqeer^2^

##### ^1^University of Turin, Italy; ^2^Al Ain University , Abu Dhabi, UAE

###### **Correspondence:** Vittorio Pace (vittorio.pace@unito.it)

*BMC Proceedings 2023*, **17(16):**P30

Cancer is a serious health problem worldwide having a high mortality rate. Cancers such as breast, colorectal, prostate and lung are amongst the most frequently diagnosed out of which lung cancer still remains the leading cause of cancer related deaths worldwide. Several challenges exist while developing anticancer drugs such as high toxicity and carcinogenicity. Also the fact that chemotherapeutic drugs need to be administered as a combination to target multiple pathways for successful treatment cannot be ignored [1,2]. Dihaloketones are well known for their potent anticancer activity. Furthermore, the alkylating activity of these drugs could be precisely tuned by proper selection of the halogens. The planar nature of the drugs can also make them effective as intercalating agents. We employed a simple, straightforward and single step synthetic methodology for the synthesis of highly valuable dihaloketones using Grignard reagents (XMgCH2XY) [3]. A reactive nucleophilic specie was generated by the reaction of Grignard reagent with different dihalomethanes. These compounds were then reacted with a range of Weinreb amides in a highly chemoselective manner. A variety of compounds were prepared having substitutions such as cynao, nitro, alkyl, heteroaryl, trifluoromethyl and halogens on the benzene ring. Compounds having different XCH2Y (X/Y = F, Cl, Br, I) combinations were prepared in high yield for further exploration.

References

1. Zugazagoitia J, Guedes C, Ponce S, Ferrer I, Molina-Pinelo S, Paz-Ares L. Current challenges in cancer treatment. Clinical therapeutics. 2016; 7:1551-66.
2. Ames BN, Gold LS, Willett WC. The causes and prevention of cancer. Proceedings of the National Academy of Sciences. 1995; 92:5258-65.
3. Armstrong DR, García-Álvarez P, Kennedy AR, Mulvey RE, Parkinson JA. Diisopropylamide and TMP Turbo-Grignard Reagents: A Structural Rationale for their Contrasting Reactivities. Angewandte Chemie. 2010; 1:3253-6.

### P31 Evaluation of knowledge, practice, and attitude of safe medication disposal among students at the universities in the United Arab Emirates: Cross-sectional study

#### Feras Jirjees^1^, Zelal Kharaba^2^, Manal Al-Sha’rawy^3^, Hala Al-Obaidi^4^, Kawthar Kayed^2^, Yassen Alfoteih^5^, Karim El-Zu'bi^1^

##### ^1^College of Pharmacy, University of Sharjah, UAE; ^2^College of Pharmacy, Al Ain University, UAE; ^3^College of Pharmacy, University of Sharjah, UAE; ^4^College of Pharmacy and Health Sciences, Ajman University, UAE; ^5^Department of Dental Surgery, City University Ajman, UAE

###### **Correspondence:** Feras Jirjees (fjirjees@sharjah.ac.ae)

*BMC Proceedings 2023*, **17(16):**P31

Background

Pollution represents is a major problem that face human beings nowadays. Pharmaceutical products are considered as environmental contaminants because of their widespread use. Therefore, waste management activities are important, to reduce unsafe disposal of medications. The knowledge and awareness of proper drug disposal are essential for safe environment. University students especially medical and pharmacy students can play a vital role in promoting safe disposal of medication. The study aimed to assess knowledge, attitude, and practice (KAP) of disposal of expired/unused medication among medical and non-medical university students in the UAE and to explore the appropriate safe medicine disposal methods.

Method

A cross sectional questionnaire study conducted over a period of four months from April to August 2022 among three universities students at the UAE. The survey was developed and validated, and distributed using google form. The study received Ethical approval from the Research Ethics Committee at the Sharjah University.

Results

Universities students from various colleges participated in the study (n=1403). Most of the participants were female (71.63%). Around half of respondents (45.54%) were from medical and health colleges. Almost two third of participants reported disposing medications by throwing them in the garbage. Less than 17% of the respondents practiced returning unused medications to the pharmacy. The level of knowledge and practice were reported to be higher in medical colleges than non-medical colleges (P value <0.05). However, the level of knowledge about disposal of pharmaceutical products was moderate, and the level of practice was poor.

Conclusion

Although there is a variation between levels of knowledge, attitude and practice among participants in the study, most of participants were ready to contribute and dispose unused/expired drugs in appropriate way if available. There is a need for appropriate awareness and guidance of students regarding safe disposal of unused and expired medications.

### P32 Evaluation of NSAIDs use and misuse by patients with cardiovascular disease in the Lebanese community

#### Mohammad Assi, Maya Ourabi, Soha Hajj Ali, Iqbal Fahes, Fadi Hdeib

##### Lebanese International University

###### **Correspondence:** Mohammad Assi (mohammad.assi01@liu.edu.lb)

*BMC Proceedings 2023*, **17(16):**P32

Background

While a notable percentage of patients have inadequate knowledge about appropriate use of non-steroidal anti-inflammatory drugs, the aim of this study is to assess the knowledge of the Lebanese population regarding the appropriateness of NSAID use, whether they are aware of the risks and adverse events caused by their use. In addition, it aims to study the effect of demographic differences on attainment of knowledge of NSAID use, and to identify the influence of pharmacist counselling on the patient's knowledge and awareness of the risks and adverse events associated with NSAID use.

Methods

A descriptive cross-sectional study was conducted among the Lebanese population. A self-administered questionnaire was used and filled by patients. Attitudes were measured using a 5-point Likert scale. SPSS software version 25 was used to conduct data analysis. A p-value less than 0.05 was considered significant.

Results

A total of 250 participants responded to the survey. NSAIDs were mostly used for a duration of less than one month, with 39.2% of the respondents using NSAIDs daily. In more than 50% of the cases, NSAIDs were prescribed by physicians. Only 80.2% of participants were aware of NSAIDs-related adverse events, with pharmacists being the number one source of information. Overall, the majority of participants were aware of the side effects caused by NSAIDs. Blood pressure elevation was the mostly reported side effect (82.4%). Only 75.6% of the patients were counseled by their pharmacists regarding the correct dose and frequency of administration of NSAIDs. However, less than one-third of respondents stated that the pharmacist role in counseling was inadequate.

Conclusion

Patient counseling has the ability to reduce possibly improper usage and raise risk awareness. Pharmacists can play a more active role in detecting and advising patients about NSAIDs. In other words, further changes are required for increased care and the avoidance of potentially disastrous incidents.

Keywords

Nonsteroidal anti-inflammatory drugs, knowledge, counseling, adverse drug events, attitude, awareness.

### P33 Identification of new potential SHP2 inhibitors by lead optimization

#### Shaima Hasan^1^, Mohammad Ghattas^1,2^, Saad Touqeer^1^, Rose Ghemrawi^1,2^, Noor Atatreh^1,2^

##### ^1^College of Pharmacy, Al Ain University, Abu Dhabi, United Arab Emirates, 64141; ^2^AAU Health and Biomedical Research Center, Al Ain University, Abu Dhabi, United Arab Emirates, 64141

###### **Correspondence:** Mohammad Ghattas (mohammad.ghattas@aau.ac.ae)

*BMC Proceedings 2023*, **17(16):**P33

Breast cancer is one of the most abundant malignancies and the second cause of death among women having cancers [1]. Scientists have always aimed to find a new therapeutic target to enhance breast cancer's clinical outcomes. The Src homology 2-containing phosphatase 2 (SHP2) was discovered to be over-expressed in breast cancer cells [2] and developing new SHP2 inhibitors are of interest. In our previous study, validated ligand-based and structural-based virtual screening protocols were conducted against the SHP2 active site. Compound NSC57774 has shown good fitting and stability against the SHP2 active site. In line with the in-silico study, the compound has shown good activity against SHP2 and a good selectivity over SHP1 with IC50 0f 0.8 μM. In the current study, this compound with a phenoxazine scaffold was taken as the lead compound. Lead optimization was initiated to find new derivatives with better in silico activity and explore new sites in the Shp2 pocket. Phenoxazine derivatives were generated. Derivatives were redocked in the shp2 pocket, where two of them have showed better docking score than NSC57774. For a more comprehensive view, a 30 ns molecular dynamic simulation was performed for these derivatives, followed by MMGBSA scoring. Synthetic routes have been developed for derivatives that showed the best in silico activity. Later, the best derivatives will be synthesized and biologically tested against SHP2 enzyme and breast cancer cell lines, which might reveal new potential shp2 inhibitors.

References

1. Khazaei, Z.; Momenabadi, V.; Ghorat, F. Global Cancer Statistics 2018: GLOBOCAN Estimates of Incidence and Mortality Worldwide Stomach Cancers and Their Relationship with the Human Development Index (HDI) Prevalence of Risky Behaviors and Related Factors among Students of Dezful View Project Meta-Analysis View Project., doi:10.32113/wcrj_20194_1257.
2. Zhou, X.; Coad, J.; Ducatman, B.; Agazie, Y.M. SHP2 Is Up-Regulated in Breast Cancer Cells and in Infiltrating Ductal Carcinoma of the Breast, Implying Its Involvement in Breast Oncogenesis. *Histopathology* 2008, *53*, 389–402, doi:10.1111/J.1365-2559.2008.03103.X.

### P34 The eating attitude amongst outstanding and ordinary students in the college of education at Al Ain University: A cross-sectional study

#### Balkees Abuawad^1,2^, Ziyad Ellala^1^, Majed El-Saleh^1^, Ahmad Al Maslamani^1,2^

##### ^1^Al Ain University, Abu Dhabi, United Arab Emirates, UAE; ^2^AAU Health and Biomedical Research Center, Al Ain University

###### **Correspondence:** Balkees Abuawad (balkees.abuawad@aau.ac.ae)

*BMC Proceedings 2023*, **17(16):**P34

Background

Proper nutrient intake and a good nutrition attitude play critical roles in personal health in terms of improved quality of health and dietary practices.

Therefore, efforts to promote nutrition education to students and create good attitudes have never been as urgent as it seems today. So it is proper to measure the eating attitude in education college students and measure the differences between outstanding and ordinary students, this will help recognize any gaps in the education curriculum. The objective of this study is to identify and detect the level of eating attitudes among students in the College of Education at Al Ain University and determine differences in eating attitudes between outstanding and ordinary students.

Materials and methods

A sample of 83 students was selected from the Education Collage at Al Ain University in UAE (41 outstanding students, 42 ordinary students). The validated eating attitudes test-26 was used to determine eating attitudes.

This study obtained the required ethical approval from the Ethics Research Committee at AAU.

Results

The result showed that the percentage of students in danger of eating attitudes was 41%. However, there were no significant differences across the median between outstanding and ordinary.

Conclusions

This sample of students is at low risk of adverse eating attitudes. However, there is a need to conduct the study on a larger sample from different colleges of the university.

### P35 Diet quality, and objectively measured physical activity and sleep are intercorrelated with flash glucose monitoring (FGM)-measured glycemic control among children with type 1 diabetes: A mixed-methods, cross-sectional study Diet, sleep, exercise, and type 1 diabetes

#### Mariam Muayyad^1^, Salah Abusnana^2,3^, Bashair M Mussa^4^, Radwa Helal^5^, Dana N Abdelrahim^6^, Naguib H Abdelreheim^3^, Elham Al Amiri^7^, Mays Daboul^8^, Zainab Al-Abdala^9^, Nader Lessan^5^, MoezAlIslam Ezzat Faris^10^

##### ^1^Nutrition department, Al Qassimi Women’s and Children’s Hospital, Sharjah, UAE; ^2^Clinical Science Department, College of Medicine, University of Sharjah, Sharjah, UAE; ^3^Diabetes and Endocrinology Department, University Hospital Sharjah, Sharjah, UAE; ^4^Basic Medical Science Department, College of Medicine, University of Sharjah, UA; ^5^Imperial College London Diabetes Centre, Abu Dhabi, UAE; ^6^Research institute for medical and health sciences, University of Sharjah, UAE; ^7^Diabetes and Endocrinology Department, Al Qassimi Women’s and Children’s Hospital, Sharjah, UAE; ^8^Nutrition Department, Novomed Medical Centre, Dubai, UAE; ^9^Diabetes and Endocrinology Department, Al Jalila Children’s Speciality Hospital, Dubai, UAE; ^10^Department of Clinical Nutrition and Dietetics, College of Health Sciences, University of Sharjah, UAE

###### **Correspondence:** MoezAlIslam Ezzat Faris (mfaris@sharjah.ac.ae)

*BMC Proceedings 2023*, **17(16):**P35

Abstract

Aims: We examined the intercorrelation between diet quality, objectively measured physical activity (PA), sleep duration, and subjectively measured sleep quality with flash glucose monitoring (FGM)-measured glycemic control among young patients with type 1 diabetes (T1D). Methods: Following cross-sectional design, Fitbit® accelerometers were used to objectively assess PA, while the validated questionnaires Pittsburgh sleep quality index and Mediterranean diet (MD) adherence were used to subjectively assess sleep and diet quality, respectively. Glycated hemoglobin (HbA1c%) and FGM-reported glycemic control components among children with T1D were assessed as well. Results: Of the 47 participants surveyed (25 boys, 22 girls, 9.31 ± 2.88 years), the majority reported high HbA1c%, good sleep quality, intermediate daily steps (10426 steps), spent < 60 minutes/day on PA, and reported high adherence to the MD. However, only one-third of the participants reported a healthy sleep duration. Only the sleep latency was associated (P<0.05) with the Time above Range Level 2 and Time below Range Level 2 (P=0.048) components of the FGM. A positive correlation (r=0.309, P=0.035) was reported between adherence to MD and Time in Range of the FGM. Conclusions: Diet quality, PA, and sleep quality are variably intercorrelated with FGM-measured glycemic control among young patients with T1D, and suggest to be considered influential factors in FGM-monitored diabetes research on this age group.

### P36 Click synthesis of nucleoside based analogues as novel sialyltransferase inhibitors

#### Ranim Al Saoud, Tareq Abu Izneid

##### Pharmaceutical sciences, Pharmacy College, Al Ain University, Al Ain, Abu-Dhabi, UAE

###### **Correspondence:** Ranim Al Saoud (ranim.aa07@gmail.com)

*BMC Proceedings 2023*, **17(16):**P36

Sialyltransferases (SA) terminate the cell surface glycoconjugates with sialic acid. Sialylated glycoconjugates play critical roles in many biological processes, such as cell-cell recognition and immune responses. However, growing evidence suggests the involvement of sialyltransferase overexpression in cancer metastasis, chemotherapy, and radiotherapy resistance which encourages the development of novel inhibitors against this target [1]. Designing inhibitors based on the transition state of the natural donor, cytidine 5′-monophosphate N-acetylneuraminic acid (CMP-Neu5Ac) considered the most successful approach to date, yet poor cell-permeability hindered proceeding into in-vivo studies [2, 3]. The introduction of a 1,2,3-triazole linker through click synthesis as an isosteric replacement of the original phosphodiester linkage [4] can produce potent cell-permeable analogues with synthetic accessibility. This study reports the successful application of click chemistry in synthesizing new triazole-linked transition-state analogues using alkyne fragments to provide novel, potent, and cell-permeable sialyltransferase inhibitors as promising therapeutic agents for cancer and metastasis. A collection of optimized analogues were synthesized using Copper-catalysed azide on nucleosides with alkyne cycloaddition, which successfully provided novel analogues that are readily accessible and ready for further exploration.

References

1. Dobie C, Skropeta D. Insights into the role of sialylation in cancer progression and metastasis. Br J Cancer 2020 1241. 2020 Nov 4;124(1):76–90.
2. Perez SJLP, Fu CW, Li WS. Sialyltransferase Inhibitors for the Treatment of Cancer Metastasis: Current Challenges and Future Perspectives. Mol 2021, Vol 26, Page 5673. 2021 Sep 18;26(18):5673.
3. Wang L, Liu Y, Wu L, Sun XL. Sialyltransferase inhibition and recent advances. Biochim Biophys Acta. 2016 Jan 1;1864(1):143–53.
4. Montgomery AP, Skropeta D, Yu H. Transition state-based ST6Gal I inhibitors: mimicking the phosphodiester linkage with a triazole or carbamate through an enthalpy-entropy compensation. Sci Rep 7. 2017;14428.

### P37 Evaluation of knowledge, screening practice, and potential risk prevalence of breast cancer among women In UAE

#### Maram Abbas^1,2^, Mirza Baig^1^

##### ^1^Department of Clinical Pharmacy and Therapeutics, Dubai Pharmacy College for Girls, Dubai, UAE; ^2^Institute of Public Health, College of Medicine & Health Sciences, United Arab Emirates University, Al-Ain, United Arab Emirates

###### **Correspondence:** Maram Abbas (dr.maram@dpc.edu)

*BMC Proceedings 2023*, **17(16):**P37

Background

Breast cancer is considered the most dangerous cancer for women, driving the highest number of mortalities in women worldwide. According to the WHO 2020 report, breast cancer showed the highest five-year prevalence in the UAE, among other cancers. This research was conducted to assess breast cancer awareness, potential risk factors, screening approaches, screening practices, barriers that prohibited the participants from screening, attitudes to seeking medical help, and the importance of early breast cancer detection among UAE women.

Methods

A cross-sectional community-based study was conducted through a web-based validated questionnaire about different aspects of breast cancer. The questionnaire was sent through social media platforms. The eligible completed were 616 responses. Data analysis was carried out using IBM SPSS version 27.

Results

This study showed a prevalence of breast cancer of 3.1% among the study population. Most participants were aged between 25 to 45 years. Regarding Breast cancer knowledge, most of the participants, 65.8%, had moderate knowledge, 19% had poor knowledge,7.8% had very poor knowledge, and only 7.6% had good knowledge. Breast cancer screening methods were the most recognized section at a mean of 76.08%, followed by knowledge of symptoms at 66.05%, and lastly was the risk factors section at 37.3%. The most recognized risk factor was the family history of breast cancer 81.7%, while sleep disturbances 13.8% were the least known. Only 14.1% of participants did the BSE regularly, and 34.9% never did do it. About 37.1% of women aged more than 40 years had never undergone mammography. The majority of participants, 72.6%, were at moderate risk of having breast cancer. Approximately 25.32% of respondents had at least one breast cancer symptom.

Conclusion

In conclusion, most participants had an acceptable level of knowledge about breast cancer with relatively higher knowledge scores for screening methods and symptoms. Participants who received information from healthcare providers or attended awareness events had a higher level of knowledge. This study shed light on the insufficient practice of BSE among the participants and, to a lower extent, mammography. Potential risk evaluation revealed a high percentage of participants suffering from many possible risk factors, in addition to the considerable portion of the population with a likely symptom of breast cancer.

### P38 Computer-aided drug design & molecular docking studies of tranilast analogues as potential inhibitors of transforming growth factor- β receptor type 1

#### Nusaiba A Babiker^1^, Ahmed T Negmeldin^1,2,3^, Eman M El-Labbad^1,4^

##### ^1^Department of Pharmaceutical Sciences, College of Pharmacy, Gulf Medical University, Ajman, UAE; ^2^Thumbay Research Institute for Precision Medicine (TRIPM), Gulf Medical University, Ajman, UAE; ^3^Department of Pharmaceutical Organic Chemistry, Faculty of Pharmacy, Cairo University, Cairo, Egypt; ^4^Pharmaceutical Chemistry Department, Faculty of Pharmacy, Ain Shams University, Abbassia 11566, Cairo, Egypt

###### **Correspondence:** Eman M El-Labbad (dr.eman.m@gmu.ac.ae)

*BMC Proceedings 2023*, **17(16):**P38

Transforming Growth Factor- β Receptor type 1 (TGF-βR1) is a bifunctional cytokine that regulates many biological responses including normal cell development, cell proliferation and apoptosis [1]. TGF-βRI overexpression is involved in cancer pathogenesis by promoting cell proliferation, progression and metastasis. Additionally, TGF-βRI induces angiogenesis and suppresses immunological responses [2]. Generally, Tranilast was approved as an antiallergic and antiasthmatic drug. Later, Tranilast was revealed to have a TGF-βR1 inhibitory effect [3]. This research describes the design of a novel series of anthranilate derivatives having various modes of interactions with TGF-βR1 compared with Tranilast. A database of novel Tranilast analogues was generated using Fragment-Based Drug Design, Molecular Operating Environment software. Representative compounds were selected from the database and docked in the identified binding site of TGF-βR1. Several compounds showed higher binding affinity for TGF-βR1 compared with the lead compound in this work, Tranilast. Compounds with high docking scores contained a positive amine group or a negative carboxylate group interacting with Asp290 or Lys 335 respectively, in the TGF-βR1 ATP binding site, or an aromatic ring interacting with Ser287, Lys337 or Ile211. The results obtained were utilized to rationally design a second generation that overcomes the synthesis infeasibility of the first generation maintaining the functional groups essential for interaction. Therefore, three series were designed via bioisosterism of the 3,4 dimethoxyphenyl moiety, esterification or amidation of the anthranilate scaffold moiety of Tranilast. Compounds of series 2 and 3 had higher docking scores than Tranilast. They also showed some interactions with Asp351, Tyr249 and lys232 which are the key amino acids in the kinase binding pocket. Furthermore, they showed additional interactions with Gly214, Ile211, Ser287, His 283, and Lys337. Compounds of the three series are under the process of synthesis and biological evaluation.

References

1. Morikawa M, Derynck R, Miyazono K. TGF- β and the TGF-β family: Context-dependent roles in cell and tissue physiology. Vol. 8, Cold Spring Harbor Perspectives in Biology. Cold Spring Harbor Laboratory Press; 2016.
2. Zhao M, Mishra L, Deng C-X. The role of TGF-β/SMAD4 signaling in cancer. Int J Biol Sci [Internet]. 2018 Jan 12;14(2):111–23. Available from: https://pubmed.ncbi.nlm.nih.gov/29483830
3. Osman S, Raza A, Al-Zaidan L, Inchakalody VP, Merhi M, Prabhu KS, et al. Anti-cancer effects of Tranilast: An update. Biomed Pharmacother [Internet]. 2021;141:111844. Available from: https://www.sciencedirect.com/science/article/pii/S0753332221006260

### P39 Accurate aggregates anticipator: Use of machine learning in the development of drug discovery tool for predicting colloidally-aggregating molecules

#### Abdallah Abou Hajal^1,2^, Richard A Bryce^3^, Mohammad A Ghattas^1,2^

##### ^1^College of Pharmacy, Al Ain University, Abu Dhabi, 64141, United Arab Emirates; ^2^AAU Health and Biomedical Research Center, Al Ain University, Abu Dhabi, 64141, United Arab Emirates; ^3^Division of Pharmacy and Optometry, School of Health Sciences, University of Manchester, Oxford Road, Manchester, M13 9PL, UK

###### **Correspondence:** Mohammad A Ghattas (mohammad.ghattas@aau.ac.ae)

*BMC Proceedings 2023*, **17(16):**P39

Colloidal aggregates are a serious problem in drug discovery and drug development projects. Aggregators bind to various enzymes non-specifically and produce wrong results during assay screening, making them a source of false positives. As a result, they may lead to the loss of years of work, effort, and resources. The goal of this work is to build a novel software tool, Accurate Aggregates Anticipator, that can distinguish between colloidal aggregators and non-aggregators in chemical space. To build the software, initial machine learning algorithms were utilized, namely, k-Nearest Neighbor (KNN) and Support Vector Machine (SVM). In numerous applications, these algorithms have demonstrated remarkable performance in terms of response speed and classification accuracy. The software has been trained on the largest datasets available, specifically on six large experimental and computational datasets, together consisting of 83,464 aggregators and 480,954 non-aggregators. Additionally, ≥ 200 molecular descriptors were utilized for the prediction of colloidal aggregators. These resultant data were used for training and testing several models created by the machine learning KNN and SVM algorithms. We are currently at phase 1 and the preliminary prototypes of the models have shown promising results, which will be further enhanced and developed in the coming stages. The resultant tool will be accessible through an online server to help other researchers predict aggregators in the early drug discovery process in order to maximize their actual hit rates and minimize the cost associated with the testing and validation process.

### P40 Development of a novel and validated protocol for covalent docking based virtual screening against SARS-Cov-2 Mpro

#### Radwa E Mahgoub^1,2^, Mohammad A Ghattas^1,2^, Noor Atatreh^1,2^, Feda E Mohamed^3^, Bassam R Ali^3,4^, Wael Rabeh^5^

##### ^1^College of Pharmacy, Al Ain University, Abu Dhabi, 64141, United Arab Emirates; ^2^AAU Health and Biomedical Research Center, Al Ain University, Abu Dhabi, 64141, United Arab Emirates; ^3^Department of Genetics and Genomics, College of Medicine and Health Sciences, United Arab Emirates University, Al-Ain 15551, United Arab Emirates; ^4^Zayed Centre for Health Sciences, United Arab Emirates University, Al-Ain 15551, United Arab Emirates ^5^Science Division, New York University Abu Dhabi, Abu Dhabi 129188, United Arab Emirates

###### **Correspondence:** Mohammad A Ghattas (mohammad.ghattas@aau.ac.ae)

*BMC Proceedings 2023*, **17(16):**P40

COVID-19 has been the third pandemic over the course of the last 20 years, resulting from the coronavirus family. It has imposed a serious challenge on global public health due to its rapid worldwide spread. The main protease enzyme (Mpro) has a major role in the SARS-CoV-2 life cycle, with proteolytic cleavage to generate functional proteins exclusively occurs after a glutamine residue, which is unlike any human protease. This makes designing irreversible covalent inhibitors an appealing approach, due to the presumed specificity of the viral Mpro. Although many irreversible inhibitors have been reported in literature, most of these belong to peptides or peptidomimetics groups with non-druglike characters. Accordingly, in this project, we aim to explore druglike peptidomimetics as potential covalent inhibitors of Mpro, using a combined approach of structure-based and ligand-based drug design. Initially, a pharmacophore was constructed using previously discovered irreversible inhibitors. The generated pharmacophore hypotheses were validated using sensitivity and specificity parameters. Interesting results were found where the best generated hypothesis showed a four point pharmacophore query with 89.5% sensitivity and 80.8% specificity. This query has been employed as a filtration tool alongside Lipinski’s rule of five and it helped us trimming the ligand library from 40,000 to 12,000. Subsequently, ligands were categorized based on their warheads and they were covalently docked into the corresponding Mpro structure (that is co-crystallized with a similar warhead). The resulting top hits were redocked using a more precise covalent docking mode, and were further evaluated using visual inspection and comparison with non-covalent XP docking. Shortlisted hits were purchased and will be experimentally tested using an in-house validated enzyme assay. Discovered hits by this work can inform us about new Mpro inhibitors with more druglike scaffolds.

### P41 Evaluating pharmacists’ practice and ability to recognize medication errors among self-medicating patients in the UAE – A simulated patient study

#### Samaa Fadda^1,2^, Akram Ashames^1,2^, Abdullah Hassan^1,2^, Ammar Jairoun^3^

##### ^1^College of Pharmacy and Health Sciences, Ajman University, Ajman, UAE; ^2^Medical and Bio-Allied Health Sciences Research Centre, Ajman University, UAE; ^3^Health and Safety Department, Dubai Municipality, Dubai, UAE

###### **Correspondence:** Samaa Fadda (samaa_fada@yahoo.com), Akram Ashames (a.ashames@ajman.ac.ae)

*BMC Proceedings 2023*, **17(16):**P41

Background

Increased public awareness, experience, and access to medical knowledge through various resources made people more comfortable and confident with self-medication. Consequently, drug research developed extensively to formulate safer and more tolerable drugs that could be sold without a prescription. While, adopting the choice of self-medication does have the advantages of giving people more control over their health and medical decisions as well as saving them time and money, such a choice also comes with relatively risky consequences. The fact that drug-drug interactions and contraindications aren’t very familiar concepts to the public makes drug combinations blindly used by patients very dangerous. This, in turn, makes things more complicated for the pharmacists who now have to pay more attention and keep a closer eye on each patient coming into the pharmacy for something as simple as a painkiller and drastically increases the necessity of patient-centered care and counselling.

Objectives

This study aims to evaluate community pharmacists’ ability to detect and act upon drug-drug interactions in various combinations of commonly purchased drugs. Furthermore, it will also evaluate their ability to determine whether the product asked for by the patient is actually contraindicated for their specific condition.

Methods

The simulated patient method was used to conduct this study. 5 simulated patients visited pharmacies in different emirates and followed a pre-planned simulation scenario in which the patient asked for 5 medications that present 3 different issues in terms of interactions, side effects, and contraindications. After completing each simulation, the volunteer filled in a data collection sheet to record all the information required for evaluating the pharmacists’ responses and practices (dispensing counselling). The results were then analyzed, and pharmacists’ practice scores were calculated and compared to determine factors that influence community pharmacists’ practice.

Results

A total of 92 pharmacists where included. The average practice score was 7.1 with a 95% confidence interval (CI) of [6.2%, 8%]. Univariate analysis showed that the emirate where the pharmacy is located, pharmacy location, pharmacist’s nationality, and counselling time were statistically significant factors affecting the practice score. More specifically, the results of statistical modelling showed that better practice was observed in medical center pharmacies, counselling time for 1 to 3 minutes, and counselling time for than 3 minutes.

Conclusion: In conclusion, the results obtained indicate the importance of continuous medical education and that there is in fact a need for developing effective and clear guidelines for the management and dispensing of OTC medications in community pharmacies.

### P42 Formulation and characterization of metronidazole-loaded nanoparticles from hydrophobically-modified maltodextrin

#### Mohammad Magramane, Mohammad F. Bostanudin, Arshad Mahmood

##### College of Pharmacy, Al Ain University, Abu Dhabi 112612, United Arab Emirates

###### **Correspondence:** Mohammad Magramane (202110145@aau.ac.ae)

*BMC Proceedings 2023*, **17(16):**P42

Background

Nanotechnology is one of several promising current approaches in developing efficient systems for intracellular delivery of therapeutic agents that can range from small molecule drugs to biomacromolecules. One of the critical problems in delivering drug intracellularly is related to the protective role of cell membranes that allow only small molecules with high lipid solubility to pass through. Biocompatibility and ease of chemical modification are attractive properties of polymeric nanoparticles and make them suitable for this approach. The derivatization of polymers with alkylglycerols can increase their hydrophobicity, thus improve the particle penetration across the membrane

Objectives

The aim of this study is to modify biocompatible and biodegradable polysaccharide named maltodextrin (that also have good drug carrier properties and offer functional groups for ease of modification) with butylglycidyl ether (BGE) and to use these materials for the formulation of nanocarriers that will be further tested for their suitability in drug delivery applications

Methods

To modify the polysaccharide, maltodextrin was treated with BGE to yield butylglyceryl derivatives. The resulting products are purified by dialysis and characterized by NMR and FT-IR spectroscopic methods. To formulate the nanoparticles, nanoprecipitation was performed. The diameter of particles is determined by Dynamic Light Scattering (DLS) and the zeta potential is determined using a Malvern Zetasizer. The nanoparticles was loaded with Metronidazole and further evaluated for drug delivery applications.

Results & Conclusion

Maltodextrin has been successfully modified with BGE, recording the degree of substitution within the 20-30% range. Nanoparticles formulated from BGE-modified maltodextrin were in the size range of 130-200 nm and exhibited acceptable negative zeta potentials that indicate good stability of the nanoformulations.

Keywords

Nanoparticles, Biopolymer, Metronidazole, Butylglyceryl maltodextrin, Drug delivery.

### P43 The putative role of autophagy in the anti-tumorigenic role of Angiotensin II Receptor Blocker Losartan in breast and lung cancer

#### Azizeh Alsayyid^1^, Rose Ghemrawi^1, 2^, Aya Al Qassem^1^, Azza Ramadan^1, 2^

##### ^1^Department of Pharmaceutical Sciences, College of Pharmacy, Al Ain University, Abu Dhabi Campus-UAE; ^2^AAU Health and Biomedical Research Center, Al Ain University, Abu Dhabi, United Arab Emirates

###### **Correspondence:** Azza Ramadan (azza.ramadan@aau.ac.ae)

*BMC Proceedings 2023*, **17(16):**P43

Background

According to the WHO, Cancer remains the leading cause of mortality worldwide. Incidences of breast and lung cancer were among the most common in 2020. The link between angiotensin II type I receptor and both breast and lung cancer are well documented. Given the involvement of angiotensin II type I receptor signaling in these cancers, its inhibition is a viable therapeutic option. We propose that the angiotensin receptor blocker Losartan, utilized for cardiovascular disease treatment, can be repurposed as an anti-tumorigenic drug for breast cancer and lung cancer treatment. Notably, the antitumor activity of several other angiotensin receptor blockers was mediated by the pro-death mechanism autophagy.

Objective

Our primary goals are to evaluate and confirm the potential protective role of Losartan in breast and lung cancer cell lines, and provide a putative mechanism that facilitates the protection. We propose that autophagy is the cellular mechanism responsible for Losartan's anti-tumorigenic effect.

Material and Methods

Breast cancer (MCF7, MDA-MB-231) and lung cancer (H292, A549) cell lines were incubated with or without Losartan (0-800 uM). MTT assay was utilized to assess survival, while scratch comb assay was used to assess migration. Autophagy protein expression will be evaluated via western blotting for the markers LC3A/B, ATG7 and p62.

Results:

Our preliminary findings showed that Losartan decreased viability in H292 and MCF-7 cell lines. Also, Losartan (400 uM) reduced migration in all cell lines except MDA -MB-231 cells. It is expected that protein expression of ATG7 and LC3 will increase while p62 will decrease, indicating autophagy activation.

Conclusion

Preliminary data suggest that Losartan is antitumorigenic in breast and lung cancer cell lines. The findings could provide clinicians with a pre-clinical proof of concept and scientifically validated research-based evidence which can support Losartan repurposing as anti-cancer therapeutic.

### P44 Investigations of amphiphilically-modified chitosan nanoparticle-based hydrogel containing naproxen for drug delivery applications

#### Nour Sammani^1,2^, Mohammad F. Bostanudin^1,2^

##### ^1^College of Pharmacy, Al Ain University, Abu Dhabi 112612, United Arab Emirates; ^2^Health and Biomedical Research Center, Al Ain University, Abu Dhabi, UAE

###### **Correspondence:** Nour Sammani (nour.sammani@aau.ac.ae)

*BMC Proceedings 2023*, **17(16):**P44

Background

Rheumatoid arthritis is the most frequent type of chronic inflammatory arthritis, with a significant economic, disability, and productivity impact on society. Naproxen, one of NSAIDs, is generally used to treat pain and stiffness in inflammatory diseases. The drug, however, has been associated with poor skin permeation hence, designing and investigating a novel naproxen formulation with an enhanced percutaneous permeation will likely have significant benefits.

Methods

Amphiphilic chitosan was chemically modified with butylglycerol through several steps starting with protecting chitosan amino group with phthaloyl groups, followed by alkylation reactions, and lastly the deprotection of chitosan amino group. Characterization of the modified chitosan included FT-IR, 1H-NMR spectroscopies, thermogravimetric (TGA), differential scanning calorimetry (DSC) techniques, and gel permeation chromatography. Modified chitosan was then used to formulate nanoparticles using ionotropic gelation methods, that were characterized by dynamic light scattering (DLS) using zeta-sizer Nano ZS. Drug loading and release ability of the nanoparticles were tested using naproxen as the model drug. The naproxen-loaded nanoparticles were incorporated into a hydrogel and further characterized for their stability, spreadability, and viscosity. The release and permeation studies of the dosage form were also reported.

Results

The synthesis of butylglyceryl-modified chitosan (GBE-CS) was confirmed, with a degree of substitution ranging from 30-50 %. The BGE-CS nanoparticles were found to be monodisperse with a size range from 150 to 200 nm. Naproxen loading degree in nanoparticles reached around 20%. The hydrogel system incorporating naproxen-containing nanoparticles showed acceptable attributes suitable for drug delivery applications. GBE-CS with higher degrees of substitution exhibited better permeation ability than GBE-CS with lower degrees of substitution.

Conclusion

A novel naproxen-loaded in modified-chitosan-nanoparticle was successfully formulated and incorporated into a hydrogel system with results showing unique properties of sustained drug delivery and enhanced permeation. Overall, the nano-colloidal systems warrant further investigation for use in percutaneous drug delivery.

### P45 Development of the asthma knowledge test (AKT) for assessing patient knowledge of asthma

#### Sanah Hasan, Shrouq Mahameed, Lamees Alakhras

##### College of Pharmacy and Health, Ajman University, Ajman, United Arab Emirates

###### **Correspondence:** Sanah Hasan (s.hasan@ajman.ac.ae)

*BMC Proceedings 2023*, **17(16):**P45

Objective

To develop and validate a measure of asthma knowledge that would be available for use in the Arabic language and context.

Methods

The study was conducted in one primary care and two asthma specialty clinics in the UAE. Individuals 18 years and older, who were medically diagnosed with asthma and who had good mastery of English or Arabic were included. The NAEPP was used as a framework to develop the Asthma Knowledge Test (AKT) questions. A measurement scale of “Yes”, “No” and “I don’t know” was selected. The questionnaire was pilot tested on a group of 10 patients for content clarity and comprehension. Cronbach alpha ≥ 0.7 was used to produce an internally consistent measure. Principal component analysis was used to determine the distinct areas of asthma knowledge covered by the AKT.

Results

The sample comprised 150 participants. Four components related to asthma as a common disease and its symptoms; triggers and control issues; inhalers; and beliefs and myths. AKT score average = 13.95 ± 2.77, range = 5.0-18.0. There were no effects of gender, age, or marital status, but there was a significant effect of participant level of education on AKT scores.

Conclusion

The AKT is a valid, reliable, and psychometrically tested tool available in Arabic. It is a valuable addition to the limited available tools for assessing asthma knowledge. It is simple and short which could be used in the clinic or community pharmacy to identify specific areas of patient education needing improvements.

